# How do we measure data sharing in the biomedical sciences? A measurement systematic review of biomedical data sharing-related knowledge, attitudes and practices across stakeholder groups, data types and geographies

**DOI:** 10.1136/bmjopen-2025-100314

**Published:** 2026-03-11

**Authors:** Lauren Maxwell, Priya Shreedhar, Regina Gilyan, Mirna Naccache, Robert F Terry

**Affiliations:** 1 Ecraid Foundation, Utrecht, The Netherlands; 2 Heidelberger Institut für Global Health, Heidelberg University Medical Faculty Heidelberg, Heidelberg, Germany; 3 TDR, WHO, Geneva, Switzerland

**Keywords:** Systematic Review, Psychometrics, Attitude, Knowledge, Surveys and Questionnaires, Patient Reported Outcome Measures

## Abstract

**Abstract:**

**Objectives:**

Enabling the reuse of participant-level health data is central to advancing public health and clinical practice. Measuring knowledge, attitudes and practices (KAP) related to data sharing is essential for understanding how stakeholders perceive data reuse and where further investment is needed. We conducted a measurement systematic review to identify and describe the development, scope and measurement properties of quantitative surveys assessing data-sharing-related KAP in biomedical research.

**Design:**

Systematic review using the COnsensus-based Standards for the selection of health status Measurement INstruments (COSMIN) approach.

**Data sources:**

Ovid (MEDLINE), EMBASE, CINAHL, PsycINFO and HaPI were searched for relevant surveys from 1 January 2000 to 7 April 2021. The Ovid (MEDLINE) search was updated on 30 May 2022 and 15 April 2024.

**Eligibility criteria:**

Quantitative surveys measuring knowledge, attitudes, behaviours or practices related to sharing or reusing participant-level health data were included.

**Data extraction and synthesis:**

Two independent reviewers screened studies, extracted data and, where possible, applied the COSMIN Risk of Bias checklist to assess survey measurement properties. We summarised survey scope, target populations, data types, development and measurement properties narratively. Due to substantial heterogeneity, survey findings were not compared across studies.

**Results:**

We screened 3684 title-abstracts, reviewed 104 full texts and extracted data from 72 publications representing 60 independent surveys. Most surveys originated from high-income countries and were used only once. Fewer than one-third reported pilot testing. Only six surveys provided sufficient information to apply COSMIN, and only three reported measurement properties, indicating low certainty in the available evidence.

**Conclusions:**

This is the first systematic comparison of the development and measurement properties of quantitative survey instruments assessing data-reuse KAP. Most surveys lacked rigorous development and reporting, limiting their utility for comparing KAP related to data sharing across stakeholders and settings. The review findings will inform the creation of a cross-country, cross-disciplinary question bank to support future tool development.

**PROSPERO registration number:**

CRD42021243926.

**STRENGTHS AND LIMITATIONS OF THIS STUDY**

- Two independent reviewers conducted screening, data extraction and COnsensus-based Standards for the selection of health status Measurement INstruments (COSMIN) assessments using standardised methods.
- The study applied the COSMIN Risk of Bias checklist to evaluate survey measurement properties in a structured and reproducible manner.
- Heterogeneous survey designs and limited reporting prevented the assessment of most COSMIN measurement domains.
- 11 of the 60 survey instruments could not be obtained in full, limiting the completeness of methodological appraisal.

## Introduction

 The reuse of participant-level health-related data can enhance the value of existing data by increasing sample sizes and incorporating diverse, clinically relevant sources of variation.^1^ Data sharing or other secure forms of data reuse (eg, federated learning, secure access through virtual research environments) help prevent redundant research and promote the translation of existing data into meaningful advances in clinical practice, including the development and evaluation of preventive, treatment and diagnostic measures.^2 3^ Data reuse can benefit both data providers and end users. For data providers, sharing and other forms of data reuse ensure that their contributions are fully used, leading to significant progress in healthcare research or public health.^2 3^ For end users, access to more comprehensive participant-level datasets enables more rigorous evidence synthesis,^4 5^ improved development of clinical risk prediction models^6^ and additional opportunities to build efficiencies in biomedical research and development. Understanding different stakeholders’ knowledge, attitudes and practices (KAP) related to biomedical data sharing can help inform interventions to promote data sharing.

Rigorous survey development ensures that a survey measures what it intends to measure.^7 8^ Survey development involves the precise definition of constructs, careful question wording, pretesting to assess comprehension and clarity and an analysis of measurement properties, including reliability and validity.^9^,^11^ Best practices for survey development include consulting subject matter experts, conducting cognitive interviews, pilot testing and using validated scales to enhance the survey’s accuracy and relevance.^12 13^ A survey developed in one context may not maintain the same measurement properties in another population due to cultural, linguistic or situational differences that affect how questions are understood and interpreted.^14^ Therefore, survey questions must be re-evaluated following translation into other languages or administration in a new population and measurement properties must be reassessed for each new population.

Given the importance of health data reuse to public health messaging, research and development and building trust in health systems and science, understanding how we measure KAP related to health data reuse is an important concern. In this measurement systematic review, we identified and described quantitative survey instruments designed for measuring KAP related to sharing participant-level data generated by human subjects in health-related research or the reuse of electronic medical record (EMR) data for different stakeholder groups, including investigators, healthcare professionals, ethics review committee (ERC) members, research participants and the general public. We also detailed the target population, focus areas and domains covered by these instruments. We also evaluate the methodological quality of these measurement tools using the COnsensus-based Standards for the selection of health Measurement Instruments (COSMIN) Risk of Bias checklist, which assesses survey development, measurement properties and evidence of measurement invariance or comparability across different populations or domains (eg, types of data, public health sectors).^15^ This review was conducted as part of the Coordination Mechanism for Cohorts and Trials (CoMeCT) initiative to coordinate the activities of Europe’s strategic adaptive platform trials and cohort studies on infectious diseases (IDs) with epidemic or pandemic potential by facilitating dialogues, sharing good practices, promoting collaboration and coordination across studies and supporting innovation in ID clinical research, including the coordination of health research projects to support the safe, equitable reuse of participant-level health data.^16^

## Methods

### Patient and public involvement

There was no patient or public involvement in the design, conduct, reporting or dissemination of this study.

### Study design and protocol registration

The objective of the systematic review was to assess qualitatively: (1) the target audience, core objectives and domains covered by quantitative surveys related to data sharing KAP; (2) the development of the surveys; and (3) the measurement properties of the surveys. The systematic review protocol was developed using the Preferred Reporting Items for Systematic Review and Meta-Analysis Protocols (PRISMA-P) guidelines^17^ and the Cochrane Handbook for Systematic Reviews.^18^ The SR protocol was registered in the PROSPERO database before the search was initiated (registration number CRD42021243926). We followed the revised PRISMA-COSMIN for Outcome Measurement Instruments (OMIs) guidelines for conducting and reporting systematic review findings of OMIs (online supplemental appendix table S1).^19^

### Search strategy

We searched the following electronic databases: Ovid (MEDLINE), EMBASE, Cumulative Index to Nursing and Allied Health Literature (CINAHL), Psychological Information Database (PsycINFO) and Health and Psychosocial Instruments Database (HaPI) for eligible studies from 1 January 2000 to 7 April 2021 to identify eligible studies and subsequently updated the search for Ovid (MEDLINE) on 30 May 2022 and 15 April 2024. The search strategy (online supplemental appendix table S2) included Medical Subject Headings (MeSH) and text terms related to data sharing. In keeping with the Peer Review of Electronic Search Strategies 2015 Guidelines,^20^ the systematic search strategy was developed through translating the research questions into a Patient or Problem, Intervention, Comparison and Outcome format, using Boolean and proximity operators, combining related MeSH terms, where available and free-text terms, applying limits and filters, including a filter for animal studies, tailoring the search to each database and piloting the search to ensure appropriate syntax. The search strategies were reviewed by an information scientist at Universitätsklinikum Heidelberg Library. We used snowball sampling, in which we examined the reference lists of included studies for additional studies that used the same survey instrument. We did not limit the search by geography or publication language. Citations were exported into EndNote for de-duplication and uploaded to the Covidence systematic review software for screening and extraction.

### Inclusion criteria

Eligible studies were peer-reviewed, primary research that presented or evaluated quantitative instruments that assessed KAP related to sharing or federated reuse of participant-level data from health-related studies, considering clinical-epidemiological, genomic and imaging data, whether data were anonymised, pseudonymised or reused without alteration or the reuse of EMR data. Included studies either presented data related to the application of the measurement tool or described the tool development.

### Exclusion criteria

Studies that described measurement tools focused on perceptions and actions solely related to sample sharing through biobanks or sample collections were not included. Systematic reviews, commentaries, editorials, conference presentations and other publications not representing peer-reviewed primary research were also excluded. We excluded qualitative studies on data-sharing KAP, even when they were used to develop the quantitative instrument. Quantitative tools used to measure data sharing in areas other than public health (eg, sociology, history, political science) were not included in the review.

### Data extraction and synthesis

Title-abstract and full-text screening were managed in Covidence. The title-abstract and full-text screening, as well as the application of the COSMIN criteria, were performed independently by two reviewers (PS, RG). Disagreements were resolved by consensus or by a third reviewer (LM) where agreement could not be reached. Data extraction was piloted with three articles. Online supplemental appendix table S4 includes general information related to the study/survey instrument (instrument name, location, year, public health field, whether the complete survey is available or not, languages, domains, survey administration), study population and sample size, recall period, study objective, data types covered by the survey, development of survey instrument and whether COSMIN Risk of Bias analysis was conducted or not. Online supplemental appendix table S4 shows deviations from the fields for data extraction detailed in the original protocol. Due to limited reporting, we did not extract detailed information on question approaches or the authors’ reasoning behind scale identification. The protocol called for extracting key survey definitions, such as broad consent, de-identification/anonymisation and data sharing; however, the manuscripts typically provided context-specific data-sharing scenarios or explanations for participants rather than explicit definitions, and we were unable to document the key definitions as planned.

**Figure 1:**
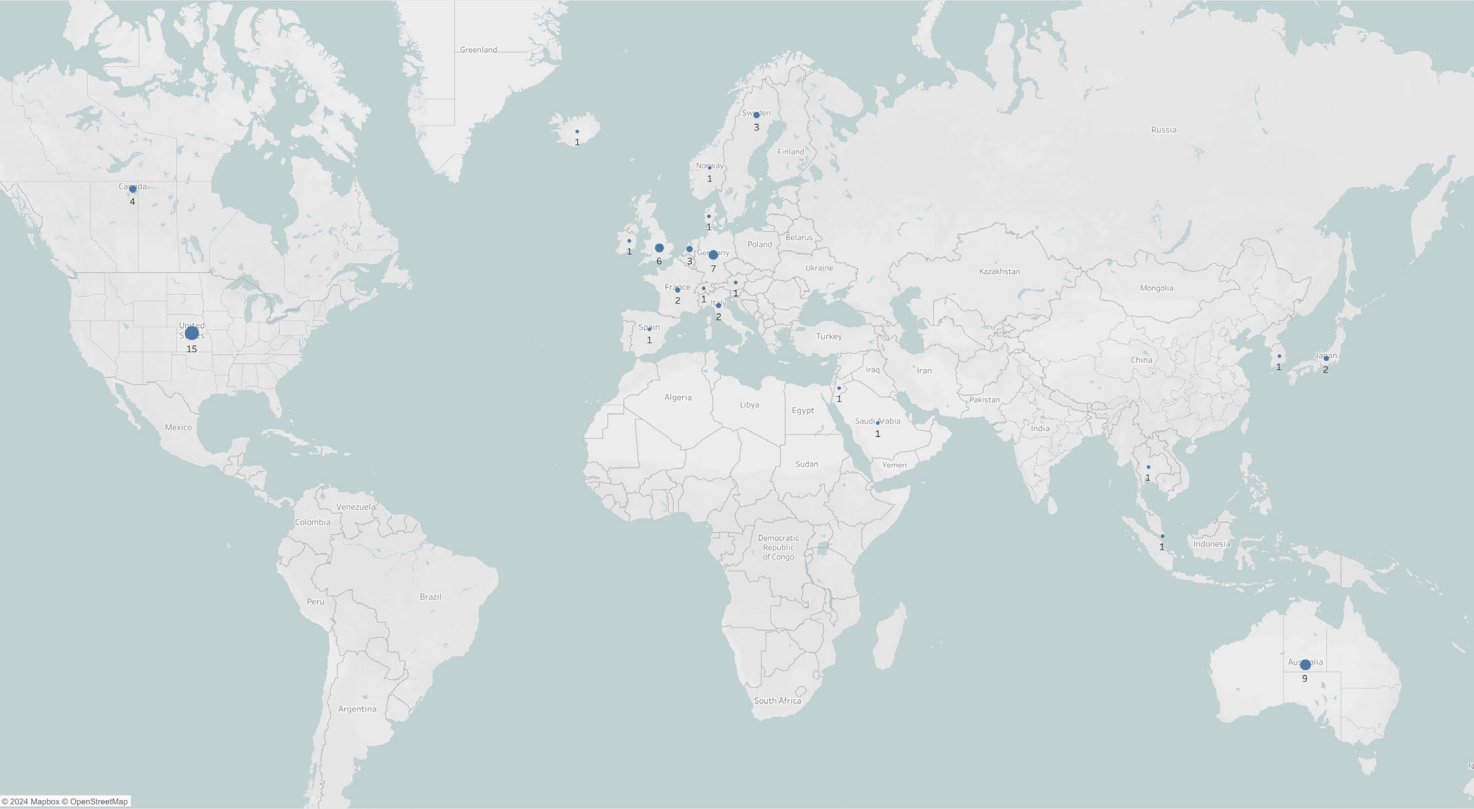
Global distribution of quantitative health data sharing knowledge, attitudes or practices-related surveys. The circle size represents the number of surveys conducted in that country.

**Figure 2:**
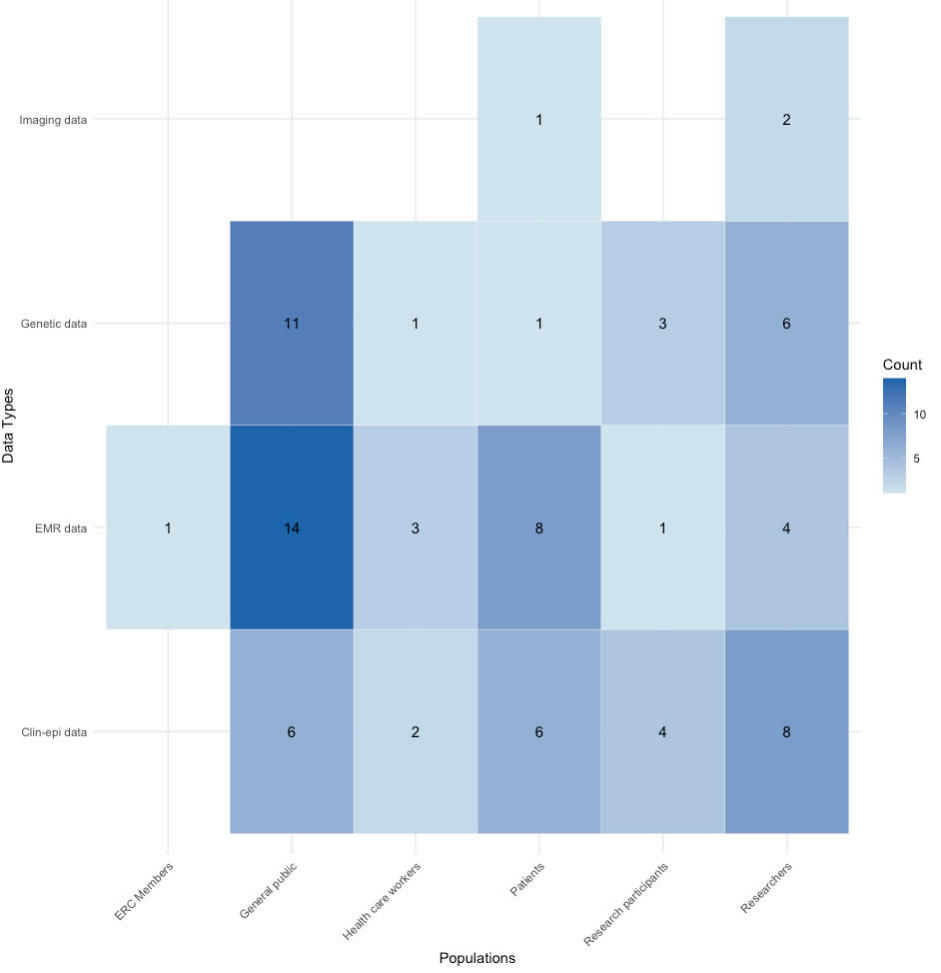
Heat map of the survey target population and data type. The darker blue corresponds to a higher count of health data sharing surveys within that combination of the population surveyed and the health data type asked about in the survey. EMR, electronic medical records.

In addition to reviewing the article text, we reviewed the survey instruments to ascertain the domains covered and to generate a question bank for future research to establish a core set of rigorously developed questions for evaluation by an expert panel as part of the first step towards establishing a core set of questions that can be recommended to groups interested in understanding different dimensions of data sharing-related KAP. When survey instruments were not provided in the publication, we emailed the authors to request them. The map presented in figure 1 was created in Tableau Cloud V.2024.3. The heat maps in figures2  3 were created using the ‘tidyverse’ library in RStudio V.2024.09.1+394.

**Figure 3:**
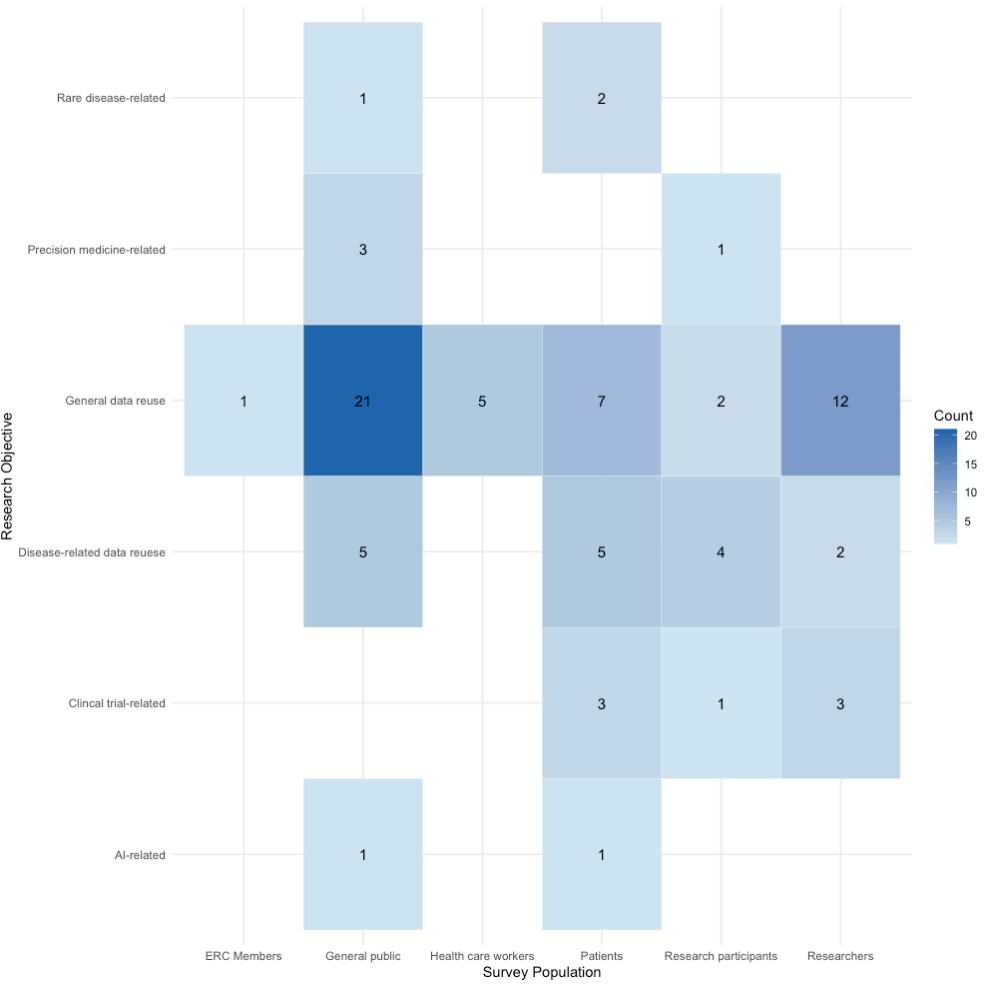
Heat map of survey target population and main survey research objectives. The darker shade of blue corresponds to a higher count of health data-sharing surveys within that combination of population surveyed and survey research objectives. AI, artificial intelligence; ERC, ethics review committee.

### Risk of bias assessment

We used the COSMIN Risk of Bias checklist for patient-reported outcome measures (PROMs)^15^ to evaluate survey instrument development and measurement properties. Publications were included regardless of whether they described the psychometric properties of the survey instruments. COSMIN includes guidance on evaluating instruments’ quality of PROM development, internal consistency, reliability, measurement error, content validity, structural validity, hypothesis testing, cross-cultural validity, criterion validity and responsiveness. The COSMIN Risk of Bias checklist is structured into 10 boxes: Box 1 assesses the quality of PROM development, and Boxes 2–10 evaluate different measurement properties. Each box guides the evaluation of specific aspects of instrument quality, ensuring a comprehensive assessment across multiple domains. Each COSMIN domain includes up to 18 items that reviewers rate on a 4-point scale (very good, adequate, doubtful and inadequate).^15^ In keeping with COSMIN guidance, the lowest item score within a domain determined the domain’s overall score.^15^ We used the COSMIN manual and consulted related literature when interpreting the criteria for assessing the quality of PROM development and each measurement property.^21^ We only conducted the COSMIN analysis when there was sufficient information on survey development or measurement properties.

## Results

**Figure 4:**
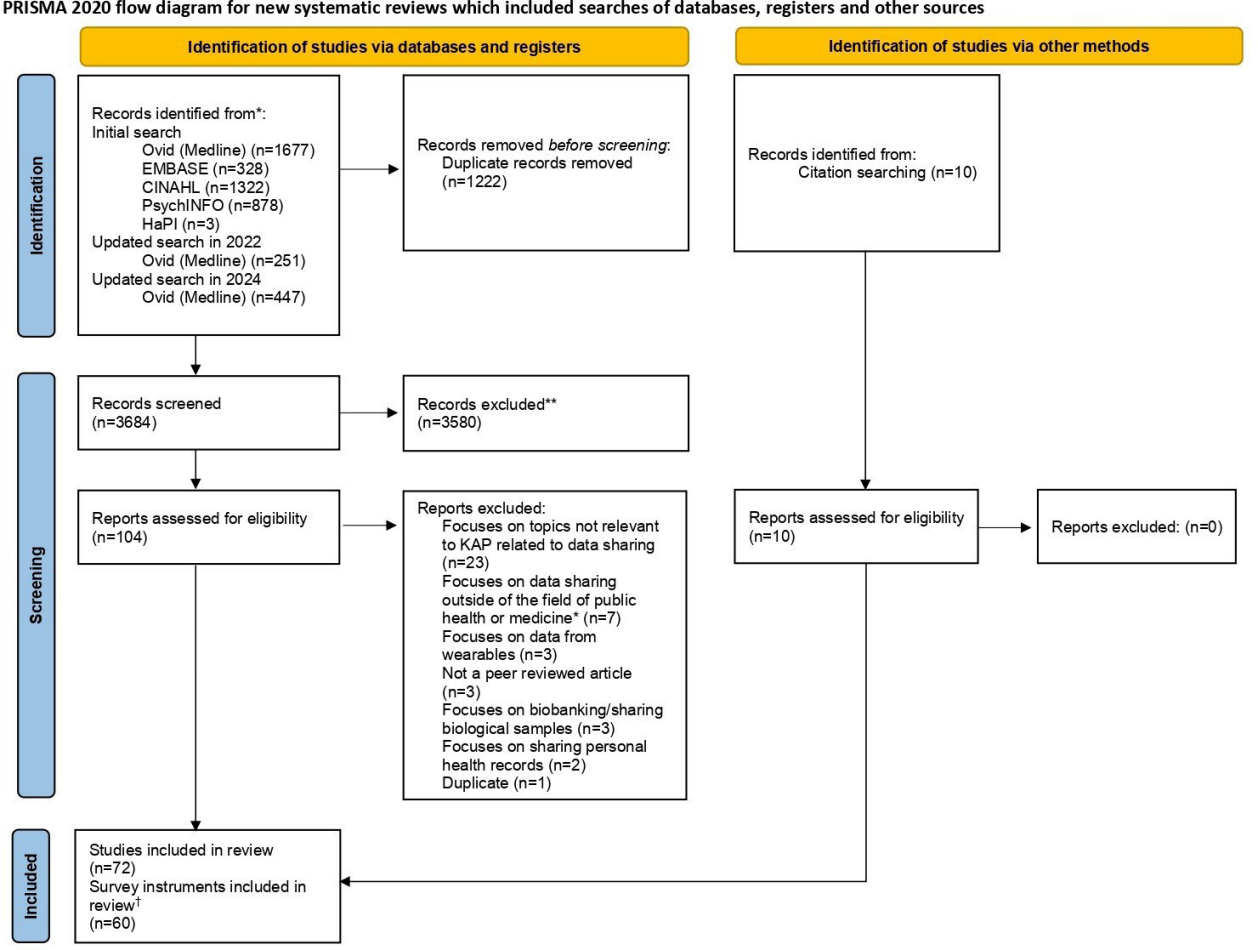
PRISMA flow diagram.^91^ *Even if several participants from medicine or health are included. †Multiple studies could cover the same survey instrument. KAP, knowledge, attitudes and practices; PRISMA, Preferred Reporting Items for Systematic Review and Meta-Analysis.

A total of 4906 records were identified through the systematic searches. After removing duplicates, 3684 records were screened by title and abstract and 104 articles were selected for full-text assessment. Of these, 62 studies identified through the systematic searches and 11 additional records found through snowball sampling were included in this review. The 72 publications represented 60 unique survey instruments. The PRISMA flow diagram presents the search and screening processes (figure 4). Please see online supplemental appendix table S3 for a list of publications excluded at the full-text stage and the reasons for their exclusion.

Of the 60 survey instruments, 24 publications (40%) did not include the entire survey in the manuscript, and we emailed the respective authors to request the missing surveys. We conducted two follow-ups over 2 months, resulting in 12 responses. Of these, 11 (18%) were shared, leaving 13 (22%) that we could not obtain for review. One author mentioned they could no longer access the survey.^22^

### Survey overview

Surveys were published between 2010 and 2024. The map in figure 1 highlights the global distribution of surveys on KAP related to data sharing. Most surveys were conducted in North America (n=19; 32%), Europe (n=11; 18%) and Oceania (n=9; 15%). Five (8%) surveys were conducted in Asia,^23^,^27^ three (5%) in the UK^28^,^30^ and two (3%) in the Middle East and North Africa (MENA) region.^31 32^ 11 surveys (18%) were conducted in multiple countries.

Close to half of the surveys (n=32; 53%) were conducted in English, six (10%) in German,^33^,^37^ two (3%) in Japanese^26 27^ and one (2%) in Dutch.^36^ 16 (27%) surveys were conducted in multiple languages. Language was not specified for 3 surveys (7%).^21 28 35^

All surveys were cross-sectional. We only found evidence that two surveys (3%) were conducted more than once and extended to different locations.^38^,^40^ Survey objectives, reported in online supplemental appendix table S4, varied widely, although all surveys asked about data-sharing attitudes and preferences.

### Survey populations

Close to half of the surveys (n=26; 43%) were limited to adult respondents: 21 surveys (35%) specified an age of 18 years and above, 1 included participants aged 20 years and above,^26^ 2 (3%) specified ages 21 years and above,^25 41^ 1 specified 35 years and above^42^ and another was limited to participants aged 20–60 years old.^27^ In an additional 27 (45%) surveys, we assumed the survey was limited to adult participants by reviewing the survey’s inclusion criteria, though the age for inclusion was not specified. The remaining three surveys (5%) included respondents aged 16 years and above,^2943^,^45^ all ages in another three surveys (5%)^28 46 47^ and 14–17 years of age in one survey.^48^

Most surveys were conducted with the general public (n=23; 38%). Patients, including those recruited from clinics and hospital waiting areas, and those with pre-existing medical conditions, were the target population in 15 surveys (25%), while researchers were the focus in 11 surveys (18%). Four surveys (7%) targeted research participants, including individuals enrolled in ongoing or recent genomic studies, biobanks or clinical trials.^49^,^53^ Three surveys (5%) involved healthcare workers,^34 54 55^ and four surveys (7%) included multiple target populations.^4656^,^58^ Later studies were more likely than earlier ones to include under-represented groups, including youth,^44^ individuals with rare diseases^43 59^ and mental health conditions.^28 30 41^

### Data types

Most surveys (n=21; 35%) focused on KAP related to the reuse of EMR data. Clinical-epidemiological (clin-epi) data was investigated in 12 surveys (20%), while 8 surveys (13%) explored both clin-epi and genetic data^22 25 27 32 43 50 60 61^ and 4 surveys (7%) addressed both clin-epi and EMR data.^38 42 54 62^ Genetic data alone was the focus of eight surveys (13%).^3133 44^,^46 57 63^ Other data types included imaging data in one survey, both EMR and imaging data in one survey^66^ and EMR and genetic data in another survey.^67^ Two surveys (3%) examined more than two data types,^24 52 53^ while two others (3%) did not explicitly specify the data types under study.^59 68^

The heat map in figure 2 shows the frequency of surveys by data type and survey population, with darker blue indicating higher counts. As shown in figure 2, EMR data was the most frequently surveyed data type among the general public (n=14; 23%) and patients (n=8; 13%),^2935 36 69^,^72^ while clin-epi data was the most frequently surveyed among researchers (n=8; 13%),^2224 32 42 61 73^,^76^ patients (n=6; 10%)^41 43 47 48 77 78^ and the general public (n=6; 10%).^25 27 38 60 62 79 80^ Genetic data was also prominent among the general public (n=11; 18%) and researchers (n=6; 10%).^22 24 32 46 61 67^ Imaging data were surveyed less frequently overall, with only small counts observed among patients and researchers, highlighting a focus on EMR, clin-epi and genetic data in health data-sharing KAP surveys.

### Survey administration

Three-quarters of the surveys were administered exclusively online. Eight surveys (13%) were paper-based only,^2932 35 51^,^53 69 70^ while three (5%) were administered via phone only.^36 49 81 82^ Three other surveys (5%) employed a combination of paper—and phone—or web-based methods,^24 71 74^ and one survey did not report the survey administration method.^78^

### Survey focus

Overall, surveys measured KAP toward health data sharing, perceptions of the risks and benefits of data sharing, motivations and barriers to data sharing, the use of data for artificial intelligence (AI) and genomic analyses and the concerns of special populations, including rare disease patients, minority youth, patients with cancer and those with chronic conditions.

Surveys primarily focused on KAP related to sharing health data in general, with 42 out of the 60 (70%) assessing KAP toward sharing health data without focusing on any particular public health field. KAP toward sharing cancer-related data was the focus of four surveys (7%),^37 57 70 77^ and toward mental health in two surveys (3%).^30 67^ Other surveys limited their inference to KAP related to rare diseases (n=2; 3%),^43 59^ biomedical research (n=1; 2%),^32^ paediatric conditions (n=1; 2%),^50^ precision medicine (n=1; 2%),^25^ sexual health (n=1; 2%),^48^ Parkinson’s disease (n=1; 2%),^47^ dental health (n=1; 2%),^42^ behavioural health (n=1; 2%),^41^ diabetes (n=1; 2%)^52 53^ and COVID-19 (n=1; 2%).^60^ One survey (2%) covered multiple areas of public health.^51^

We could not identify any patterns in the survey focus by geography. Even within the same regions, surveys examined different KAP aspects of sharing data types with varied stakeholder groups.

As shown in figure 3, the survey focus was clustered somewhat within stakeholder groups. Surveys targeting the general public predominantly examined data reuse without a specific disease or subject area focus (n=21; 35%). Surveys on general biomedical data reuse were also the most common for researchers (n=12; 20%) and patients (n=7; 12%).^35 36 69 71 72 78^ Questions on data reuse tied to specific diseases and conditions were the most common for patients (n=5; 8%)^41 47 48 70 77^ and the general public (n=5; 8%)^28 30 37 57 60 83^ and second-most frequent for research participants (n=4; 7%).^50^,^5357 83^ All the surveys for healthcare workers focused on data reuse in general.^34 46 54 55 58^ The single survey involving ERC members examined data reuse in general.^56^

Earlier studies, published closer to 2010, centred on foundational attitudes toward data sharing in the general public but expanded in later years to include more diverse populations, such as rare disease patients,^43 59^ minority youth^48^ and patients with cancer,^37 57 70 77 83^ and an increasing emphasis on genomic data sharing and ethical considerations in biobanking and clinical trial contexts. Studies from 2020 onward reflect a shift toward assessing attitudes towards the role of AI in healthcare data and ethical concerns in data reuse.^29 31^

### Domains and question types

The surveys examined various domains of data sharing and reuse, including general views, attitudes and willingness to share data. All surveys collected some type of demographic information, and the number of items per domain varied widely. Table 1 summarises the most common domains across different survey populations. Domains covered in the surveys for the general public, research participants and patients were highly similar, so these populations were grouped together in the table. Researchers were included separately in the table; however, surveys focusing on healthcare workers were too few (n=3),^34 54 55^ and surveys with multiple target populations were too varied to describe common domains meaningfully.^4656^,^58^

**Table 1:** Survey domains by population

Population	Common domains
General public, patients and research participants	Willingness to share different data types with different information users, for different purposes Previous experiences with data sharing Knowledge/awareness of repositories, clinical trials, legal aspects of data sharing, different data sharing methods, consent options Concerns about privacy, data management and perceived risks of data sharing Perceived societal benefits of data sharing Trust in various stakeholders and institutions Expectations of information (eg, return of findings)
Researchers	General attitudes toward data sharing Experiences with sharing own research data Experiences with requesting data from other researchers Perceived concerns about data reuse/data sharing Encountered barriers and facilitators of data sharing (including ethical considerations and motivations) Knowledge and experiences with regards to data management

For the general public, patients and research participants, surveys frequently assessed willingness to share different data types with various information users for different purposes. Many surveys also investigated prior experiences with data sharing, knowledge and awareness of repositories and clinical trials, legal aspects of data sharing and consent options. Concerns about privacy, data management and perceived risks were common themes, alongside questions about the perceived societal benefits of data sharing. Trust in stakeholders and institutions and participants’ expectations regarding the return of information (eg, incidental findings) were also widely explored.

For researchers, surveys often examined general attitudes toward data sharing and experiences with sharing their own research data or requesting data from other researchers. Many surveys focused on concerns about data reuse and sharing and encountered barriers and facilitators, including ethical considerations and motivations. Additionally, some surveys assessed researchers’ knowledge and experiences regarding data management.

Question types included Likert, multiple-choice and open-ended. Additionally, four surveys (7%) featured vignette or scenario-based questions,^63 65 69 86^ while another three (5%) used discrete choice experiments (DCEs).^38^,^4062^ Later studies were more likely to use sophisticated methodologies, including DCE and scenario-based surveys, to assess nuanced preferences and trade-offs in data sharing.

### Recall period

In 23 (38%) of the surveys, respondents were asked about their future preferences regarding data sharing. Four surveys (7%) focused on current views on data sharing,^24 35 49^ while one (2%) inquired about past experiences with data sharing.^68^ A large portion of the surveys (n=24; 40%) employed a mixed recall period, combining questions about past experiences and future preferences regarding data sharing. Six surveys (12%) were limited to DCEs or scenarios/vignettes, with no recall period.^38^,^4062 65 69 86^ In one survey (2%) where the recall period was applicable, the recall period was not reported and we could not verify this information because we did not have access to the complete survey.^72^

### Survey development

We identified documentation of the survey development process for 41 (68%) surveys. Of these, 27 surveys (66%) reported implementing multiple survey development activities, and 14 (34%) reported using only one development method. 16 surveys (27%) reported piloting the survey before administration, either alone (n=4; 7%)^42 61 62 68^ or in addition to other survey development activities (n=12; 20%). Four surveys (7%) conducted cognitive interviews,^38^,^4057 70 80 83 87^ and eight (13%) conducted focus group discussions^2538 43 46 51 71 79^,^82 87^ in addition to other activities for survey development. Two surveys (3%) conducted literature reviews alone,^24 32^ and 18 (30%) conducted literature reviews and other survey development activities. 4 surveys (7%) were developed solely from pre-existing surveys,^36 65 67^ and 10 (17%) used pre-existing surveys alongside other development methods.^25 37 41 43 49 64 70 76 79 82^ 10 surveys (17%) reported conducting survey pretesting alongside other development activities.^2834 41 44^,^46 56^ Other methods used for survey development included subject matter expert input, including workshops and webinars with experts (n=16; 27%), patient advisory group input (n=3; 5%),^43 47 88^ patient/public panel input (n=5; 8%),^29 30 39 40 52 53 70^ Delphi exercise (n=1; 2%)^43^ and using pre-developed guidelines from the Global Alliance for Genomics and Health Data Use Ontology (n=1; 2%).^64^ A DCE-based survey also reported conducting a nominal group technique, a think-aloud exercise and an online ranking survey.^38 80 87^ We could not identify any documentation of the methods used to develop about one-third of the surveys (n=19; 32%).

### COSMIN analysis

The results of the COSMIN Risk of Bias checklist analysis are reported in table 2. Only six (10%) of the surveys provided sufficient detail on survey development to be evaluated against the COSMIN checklist’s criteria.^2938^,^40 44^ To be evaluated per the COSMIN criteria, we required that at least Box 1 be completed, including evidence of a PROM design and a cognitive study or pilot testing. Brief mentions of pilot testing or cognitive interviews without sufficient detail were not considered adequate. The six surveys assessed had different approaches to survey development. One conducted a pilot study alone,^29^ two conducted pilot studies in addition to cognitive interviews to improve question clarity and respondent understanding^38 57 80 83 87^ and one conducted a pilot study via cognitive interviews.^39 40^ Another survey conducted a pilot study and asked subject matter experts about the relevance and comprehensiveness of the survey items (content validity).^44 45^ One study assessed patients’ comprehension of the survey and subject matter experts’ perceptions of the survey’s relevance and comprehensiveness (content validity), in addition to conducting a reliability assessment and a pilot study.^46^ None of the studies reported all the measurement properties required by the COSMIN Risk of Bias checklist. Studies that did report measurement properties lacked sufficient detail to fully satisfy the COSMIN checklist’s criteria. Only three of the six surveys reported any measurement properties. One survey addressed reliability,^46^ and all three addressed content validity.^3844^,^46 80 87^

**Table 2:** Rating and quality of the evidence for measurement properties using the COSMIN checklist^15^

PROM	Patient and public views on data sharing for AI research^29^		Public preferences for digital health data sharing^38 80 87^		Participant issues and expectations project^57 83^		Attitudes toward sharing genomic data^46^		Your DNA, your say^44 45^		The relative importance of different attributes in influencing preferences of data sharing^39 40^	
	Rating	QoE	Rating	QoE	Rating	QoE	Rating	QoE	Rating	QoE	Rating	QoE
PROM design	D	Low	A	Moderate	V	High	D	Low	I	Low	D	Low
Pilot/cognitive interview	D	Low	D	Low	D	Low	D	Low	D	Low	D	Low
Content validity												
Relevance (patients)	–	–	V	High	–	–	–	–	–	–	–	–
Comprehensiveness (patients)	–	–	V	High	–	–	–	–	–	–	–	–
Comprehensibility (patients)	–	–	–	–	–	–	–	–	–	–	–	–
Relevance (professionals)	–	–	A	Moderate	–	–	–	–	D	Low	–	–
Comprehensiveness (professionals)	–	–	A	Moderate			D	Low	D	Low	–	–
Structural validity	–	–	–	–	–	–	–	–	–	–	–	–
Internal consistency	–	–	–	–	–	–	–	–	–	–	–	–
Cross-cultural validity	–	–	–	–	–	–	–	–	–	–	–	–
Measurement invariance	–	–	–	–	–	–	–	–	–	–	–	–
Reliability	–	–	–	–	–	–	I	Low	–	–	–	–
Measurement error	–	–	–	–	–	–	–	–	–	–	–	–
Criterion validity	–	–	–	–	–	–	–	–	–	–	–	–
Construct validity	–	–	–	–	–	–	–	–	–	–	–	–
Responsiveness	–	–	–	–	–	–	–	–	–	–	–	–

A, adequate; AI, artificial intelligence; COSMIN, COnsensus-based Standards for the selection of health Measurement Instruments; D, doubtful; I, inadequate; PROM, patient-reported outcome measures; QoE, quality of evidence; V, very good.

## Discussion

In this systematic review, we identified, described and compared the measurement properties of survey instruments used to estimate KAP related to biomedical data sharing. Survey instruments focused on attitudes toward data sharing, knowledge and awareness of data-sharing practices, perceptions of benefits and risks, motivations and barriers and the use of data for AI and genomic research. Surveys addressed issues of privacy, consent and trust. They explored the perspectives of the general public, patients with specific health conditions (eg, cancer, rare diseases) and researchers on EMR, clin-epi, genetic and imaging data. The clustering of the thematic focus of data-sharing KAP-related surveys underscores how local health policies, cultural factors, data-sharing and reuse infrastructure and local priorities influence the survey focus and scope.

Most surveys were conducted in high-income countries. Future surveys should aim for more diverse populations, including underrepresented regions in MENA, Asia, Africa and Latin America and the Carribean (LAC), to address gaps in geographical and demographic representation. Surveys could also consider incorporating additional populations that are often excluded from biomedical research, including children, pregnant women, or incarcerated, indigenous, displaced and migrant populations, to consider further sources of heterogeneity in data sharing and reuse preferences.

We were not able to review 11 of the 60 surveys. Survey authors should provide full versions of their surveys, linked to the publication, to enhance transparency and enable a more thorough evaluation of the survey instruments. We could not find documentation of the development process for close to one-third of the surveys. Only 16 of the 60 questionnaires were pilot tested. The lack of pilot testing means respondents may not have understood the questions, and investigators may not have identified validity and reliability issues before implementing the survey.

Most instruments were applied in the USA or Europe and were only available in English. We identified very few surveys conducted in low- and middle-income countries, suggesting an inequitable distribution of efforts to measure biomedical data sharing-related KAP. No surveys were identified for China or India, the world’s most populous countries, which may be related to our search strategy, which did not include Chinese-language biomedical databases. Administering surveys in multiple languages, as was done for a few surveys conducted in Europe, North America and Asia, should be encouraged to increase accessibility and representation across different linguistic groups. However, it would be necessary to first test respondents’ understanding and measurement of the survey’s properties in the new languages through cognitive interviewing and measurement invariance analysis.^89^

Given that three-quarters of surveys were administered exclusively online, researchers could explore alternative methods to ensure inclusivity of populations with limited internet access, such as paper-based surveys or phone interviews. Most surveys focused on reusing EMR, clin-epi or genomic data. Additional research could explore KAP related to other types of health data, including patient-generated health data, microbiome data and vaccination data. Given that over one-third of surveys assessed future data-sharing preferences, additional research could employ longitudinal designs or repeated cross-sectional surveys to explore changes in data-sharing attitudes and behaviours over time.

We used the COSMIN Risk of Bias checklist to systematically assess the methodological quality of research on the measurement attributes of health-related PROMs.^15^ While 72 studies representing 60 instruments were included in the review, we could apply COSMIN only to the 6 instruments for which survey development was adequately reported.^2938^,^40 44^ We evaluated the measurement properties of three of those six, as the other three did not report any measurement properties.^3844^,^46 80 87^ According to the COSMIN Risk of Bias checklist, all six studies had a high risk of bias, indicating poor methodological quality. The only measurement properties reported were reliability and content validity. No longitudinal studies were available, so criterion validity and responsiveness could not be evaluated. No studies conducted hypothesis testing or addressed measurement invariance.

COSMIN covers survey design, methodology and measurement properties, including content validity, reliability, internal consistency, cross-cultural validity, responsiveness and content and structural validity, among other properties.^15^ There could be several reasons why we could not identify sufficient information to apply COSMIN to all but six surveys, and why only three reported any measurement properties. First, KAP related to data sharing and reuse may not be seen as central to patient health, so the same rigour expected when developing quantitative PROM measures may not be required. An additional constraint is that most data-sharing-related KAP instruments are not designed as reflective measurement tools with clearly defined constructs or multi-item scales suitable for psychometric evaluation. COSMIN can be applied to any self-reported instrument in principle, including surveys on KAP; however, in practice, the conceptual heterogeneity of items, the absence of underlying scale structures and the formative nature of many KAP domains mean that most instruments did not meet the conditions necessary for COSMIN assessment. Furthermore, research on data-sharing KAP may be underfunded, leading to studies that lack sufficient support for rigorous survey development and testing. Some studies may have undertaken rigorous survey development or assessed survey measurement properties but did not report these steps. Finally, some argue that the COSMIN criteria are too complicated to evaluate and not reproducible.^90^ Reviews using the COSMIN checklist often yield inconsistent conclusions and lack evidence to support their recommendations, raising concerns about the checklist’s reliability and validity.^90^ Additionally, many reviewers lack the necessary expertise in psychometrics and instrument development, undermining the quality of their evaluations. As modern PROM development remains in its early stages, the limited availability of high-quality PROMs reduces the necessity for complex reviews. Still, challenges with the application of the COSMIN criteria would not have affected our finding that even minimal information on survey development and properties was not reported.

Authors should provide more detailed descriptions of their survey development methods to enable an assessment of the quality of the development process. Researchers should consider using a combination of methods for survey development, including pilot testing, cognitive interviews and expert input, to enhance the validity and reliability of their instruments.^7 9 10^ To strengthen the reliability of survey results, researchers should consider validating their instruments through qualitative and quantitative approaches, such as focus groups and statistical reliability testing, before final implementation.^7 9 10^ Reviewers should ensure that publications related to survey development and administration provide sufficient information to enable readers to apply the COSMIN Risk of Bias checklist to evaluate survey design and measurement properties, such as reliability and content validity. Future studies can produce more reliable and generalisable data on data-sharing attitudes and preferences by addressing these gaps and improving survey instruments’ transparency, methodology and inclusivity.

### Strengths and limitations

This systematic review provides a comprehensive overview of the development, scope and measurement properties of quantitative survey instruments for measuring KAP related to sharing participant-level health-related data. This is the first study to compare quantitative surveys for assessing KAP related to biomedical data reuse. A strength of this systematic review is that it uses the COSMIN Risk of Bias checklist to provide a structured, consistent way to assess the survey instruments and the quality of their measurement properties.

This study also has some limitations. While we tried to obtain the original surveys from the included studies, we were unable to obtain complete surveys from 11 of the 60 included studies. While it is unlikely, given that we used snowball sampling to identify related studies for each included publication, some groups may have completed survey development or validation studies that were not reported in the studies we identified for each survey. Most COSMIN criteria could not be evaluated because of insufficient data reported in publications or because the surveys were not rigorously developed. We repeated the search in OVID (MEDLINE) in 2023 and 2024 to ensure it was up to date, but we did not repeat it in the additional three databases due to limited funding for this work. Lastly, we did not compare the findings of the quantitative surveys because of the high level of heterogeneity in survey objectives and designs and because that was out of scope for this measurement systematic review.

### Recommendations

Our findings suggest that further investment is needed to support the development of data reuse KAP-related surveys that are valid and reliable to facilitate longitudinal analyses that can identify significant changes in data reuse KAP over time and across geographies, demographic factors (eg, age, gender, race, ethnicity), stakeholder groups (eg, healthcare staff, research staff, research participants, patients, ERC members), data types and disease areas.

Developing a conceptual framework, item generation, item refinement through cognitive interviews, pilot testing, item reduction using statistical techniques and psychometric evaluation, which includes reliability, validity, responsiveness testing, cultural adaptation if needed, refinement based on evaluation results and continuous evaluation of performance, including measurement invariance testing, is needed to foster a rigorous survey development process for understanding biomedical data sharing KAP. This procedure guarantees that the quantitative survey has the desired psychometric qualities for its intended use, is easily understood by the target population and accurately measures the intended constructs.

KAPs regarding the sharing of participant-level health-related data likely vary across geography, stakeholder groups, age and the public health field. For example, researchers who collect data likely have different motivations and barriers to sharing than ERC members who evaluate proposals that require data sharing, or research participants who are asked to consent to the use of their data for future research. Understanding how KAP relates to data sharing varies across stakeholder groups, contexts and geographies and across the public health field, can help policymakers and researchers understand how to address political, ethical, administrative, regulatory and legal barriers to data sharing. Future research could also consider how attitudes towards biomedical data and sample sharing vary. While we cannot recommend a survey to carry forward and test across different populations, the 49 complete surveys that we reviewed and collected in this review can serve as a question bank to use as a first step for the development of a quantitative survey to measure data reuse-related KAP within or across stakeholder groups and geographies.

## Conclusion

As data reuse is increasingly prioritised, policymakers and health systems must understand KAP related to biomedical data reuse across stakeholder groups and modalities (eg, data transfer, trusted research environments, federated data reuse). Almost no biomedical data-sharing-related survey instruments were reused in a novel population or more than once. Without the ability to evaluate scale validity and reliability, we cannot suggest scales for translation into novel contexts or for repeated use within the same populations over time to understand temporal or population-related changes in data-sharing KAP. That said, findings from this comprehensive overview can be used to generate a question bank to create a rigorously developed instrument.

Within the CoMeCT project, understanding stakeholder KAP related to biomedical data reuse is essential to promote efficient, comprehensive and collaborative research that spans domains and populations and becomes especially important in the research response to emerging pathogens. CoMeCT partners will prioritise follow-up research to support the rigorous development of a quantitative survey for assessing data reuse KAP that can be applied across disease contexts and stakeholder groups using the question bank established through this review, communication with subject matter experts and experts in survey development and following best practice in survey design and evaluation.

## Supplementary material

10.1136/bmjopen-2025-100314 online supplemental file 1

10.1136/bmjopen-2025-100314 online supplemental file 2

10.1136/bmjopen-2025-100314 online supplemental file 3

10.1136/bmjopen-2025-100314 online supplemental file 4

## Data Availability

All data relevant to the study are included in the article or uploaded as supplementary information.
