# Supplementary material for: How do we measure data sharing in the biomedical sciences? A measurement systematic review of biomedical data sharing-related knowledge, attitudes and practices across stakeholder groups, data types and geographies
Source: BMJ Open. 2026 Mar 11;16(3):e100314. doi: 10.1136/bmjopen-2025-100314 (PMC12983744; doi:10.1136/bmjopen-2025-100314)
Supplement: online supplemental file 2 [file bmjopen-16-3-s002.pdf]

Table 1. Ovid(Medline) Search Strategy 07 April 2021- 30 May 2022

#1	(data adj2 (shar* OR exchange* OR secondary use OR interchange* or access or link*)).ti
#2	(data adj2 (knowledge or attitude* or practice* or willing* or belief* or value* or perception* or expectation*)).ti
#3	(((broad or blanket or wide or open) adj2 consent) or secondary use* or future use*).ti
#4	exp Surveys/ Or exp Questionnaires/
#5	exp Educational Measurement/ or Exp self-assessment/ or Exp psychometrics/
#6	(questionnaire* or survey* or instrument or measure* or tool or inventory or psychometrics or reliability or validity or measure* invariance or understand* or evaluat*).ti,ab.
#7	or/1-3
#8	or/4-6
#9	7 and 8
#10	exp animals/ not humans.sh.
#11	9 not 10
#12	11 not ((editorial or commentaries or systematic review or letters).de. or (editorial or commentaries or systematic review or letters).pt.)
#13<br>#14	limit 12 to yr="2000 -Current"<br><br>Limit 13 to yr=”2021-current’’

OvidMedline – Search 07 April 2021

#1	(data adj2 (shar* OR exchange* OR secondary use OR interchange* or access or link*)).ti
#2	(data adj2 (knowledge or attitude* or practice* or willing* or belief* or value* or perception* or expectation*)).ti
#3	(((broad or blanket or wide or open) adj2 consent) or secondary use* or future use*).ti
#4	exp Surveys/ Or exp Questionnaires/
#5	exp Educational Measurement/ or Exp self-assessment/ or Exp psychometrics/
#6	(questionnaire* or survey* or instrument or measure* or tool or inventory or psychometrics or reliability or validity or measure* invariance or understand* or evaluat*).ti,ab.

#7	or/1-3
#8	or/4-6
#9	7 and 8
#10	exp animals/ not humans.sh.
#11	9 not 10
#12	11 not ((editorial or commentaries or systematic review or letters).de. or (editorial or commentaries or systematic review or letters).pt.)
#13	limit 12 to yr="2000 -Current"

Ovid Embase

#1	(data adj2 (shar* OR exchange* OR secondary use OR interchange* or access or link*)).ti
#2	(data adj2 (knowledge or attitude* or practice* or willing* or belief* or value* or perception* or expectation*)).ti
#3	(((broad or blanket or wide or open) adj2 consent) or secondary use* or future use*).ti
#4	exp Surveys/ Or exp Questionnaires/
#5	exp Educational Measurement/ or Exp self-assessment/ or Exp psychometrics/
#6	(questionnaire* or survey* or instrument or measure* or tool or inventory or psychometrics or reliability or validity or measure* invariance or understand* or evaluat*).ti,ab.
#7	or/1-3
#8	or/4-6
#9	7 and 8
#10	exp animals/ not humans.sh.
#11	9 not 10
#12	11 not ((editorial or commentaries or systematic review or letters).de. or (editorial or commentaries or systemat[illegible]

#13	limit 12 to yr="2000 -Current"

APA Psycinfo

#1	TI ((data N2 (shar* OR exchange* OR secondary use OR interchange* or access or link*)))
#2	TI ((data N2 (knowledge or attitude* or practice* or willing* or belief* or value* or perception* or expectation*)))
#3	TI ((((broad or blanket or wide or open) N2 consent) or secondary use* or future use*))
#4	DE ("Surveys" OR "Consumer Surveys" OR "Mail Surveys" OR "Online Surveys" OR "Telephone Surveys" OR "Questionnaires" OR "General Health Questionnaire")
#5	DE (("Educational Measurement+") OR ("Self Assessment") OR ("Psychometrics"))
#6	TI ((questionnaire* or survey* or instrument or measure* or tool or inventory or psychometrics or reliability or validity or measure* invariance or understand* or evaluat*)) OR AB ((questionnaire* or survey* or instrument or measure* or tool or inventory or psychometrics or reliability or validity or measure* invariance or understand* or evaluat*))
#7	S1 OR S2 OR S3
#8	S4 OR S5 OR S6
#9	S7 AND S8
#10	TI (((animal* OR canine* OR dog* or feline* OR hamster* OR lamb* OR mice OR mouse OR monkey* OR murine OR pig* OR piglet* OR porcine OR primate* OR rabbit* OR rat* OR rodent* OR sheep* OR frog* OR worm* OR trematode))) NOT TI (((human* OR patient*))))
#11	S9 NOT S10
#12	S11 NOT ((editorial or commentaries or systematic review or letters))
#13	limit S12 to Published Date: 20000101-20211231

CINAHL

#1	TI ((data N2 (shar* OR exchange* OR secondary use OR interchange* or access or link*)))
#2	TI ((data N2 (knowledge or attitude* or practice* or willing* or belief* or value* or perception* or expectation*)))
#3	TI ((((broad or blanket or wide or open) N2 consent) or secondary use* or future use*))

#4	MH ("Surveys+") OR MH ("Questionnaires+")
#5	MH ("Educational Measurement+") OR MH ("Self Assessment") OR MH ("Psychometrics")
#6	TI ((questionnaire* or survey* or instrument or measure* or tool or inventory or psychometrics or reliability or validity or measure* invariance or understand* or evaluat*)) OR AB ((questionnaire* or survey* or instrument or measure* or tool or inventory or psychometrics or reliability or validity or measure* invariance or understand* or evaluat*))
#7	S1 OR S2 OR S3
#8	S4 OR S5 OR S6
#9	S7 AND S8
#10	TI (((animal* OR canine* OR dog* or feline* OR hamster* OR lamb* OR mice OR mouse OR monkey* OR murine OR pig* OR piglet* OR porcine OR primate* OR rabbit* OR rat* OR rodent* OR sheep* OR frog* OR worm* OR trematode))) NOT TI (((human* OR patient*))))
#11	S9 NOT S10
#12	S11 NOT ((editorial or commentaries or systematic review or letters))
#13	limit S12 to Published Date: 20000101-20211231

HaPi

#1	secondary use.ti
#2	Data sharing.ti
#3	Data exchange
#4	Broad consent
#5	Blanket consent
#6	Future use
#7	Open consent
#8	1-7/or
#9	Limit 2000-2020
