# Supplementary material for: How do we measure data sharing in the biomedical sciences? A measurement systematic review of biomedical data sharing-related knowledge, attitudes and practices across stakeholder groups, data types and geographies
Source: BMJ Open. 2026 Mar 11;16(3):e100314. doi: 10.1136/bmjopen-2025-100314 (PMC12983744; doi:10.1136/bmjopen-2025-100314)
Supplement: online supplemental file 3 [file bmjopen-16-3-s003.pdf]

Appendix Table S3. List of excluded studies along with reasons for exclusion

Exclusion reason	Citation	Title	Authors	Published Year	DOI
Duplicate	Richter 2021	**Correction: Secondary research use of personal medical data: attitudes from patient and population surveys in The Netherlands and Germany**	Richter, Gesine; Borzikowsky, Christoph; Lesch, Wiebke; Semler, Sebastian C.; Bunnik, Eline M.; Buyx, Alena; Krawczak, Michael	2021	https://dx.doi.org/10.1038/s41431-021-00971-1
Focuses on biobanking/ sharing biological samples	Smith 2016	**Conducting a large, multi-site survey about patients' views on broad consent: challenges and solutions**	Smith, Maureen E.; Sanderson, Saskia C.; Brothers, Kyle B.; Myers, Melanie F.; McCormick, Jennifer; Aufox, Sharon; Shrubsole, Martha J.; Garrison, Nanibaá A.; Mercaldo, Nathaniel D.; Schildcrout, Jonathan S.; Clayton, Ellen Wright; Antommaria, Armand H. Matheny; Basford, Melissa; Brilliant, Murray; Connolly, John J.; Fullerton, Stephanie M.; Horowitz, Carol R.; Jarvik, Gail P.; Kaufman, Dave; Kitchner, Terri	2016	10.1186/s12874-016-0263-7
	Alrabadi 2019	**Jordanians' Perspectives On Open Consent In Biomedical Research**	Alrabadi, Nasr; Makhlouf, Hanin; Khabour, Omar F.; Alzoubi, Karem H.	2019	https://dx.doi.org/10.2147/RMHP.S217209
	DeVries 2016	**Understanding the Public's Reservations about Broad Consent and Study-By-Study Consent for Donations to a Biobank: Results of a National Survey**	De Vries, Raymond Gene; Tomlinson, Tom; Kim, Hyungjin Myra; Krenz, Chris; Haggerty, Diana; Ryan, Kerry A.; Kim, Scott Y. H.	2016	https://dx.doi.org/10.1371/journal.pone.0159113
Focuses on data from wearables	Karampela 2019	**Connected Health User Willingness to Share Personal Health Data: Questionnaire Study**	Karampela, Maria; Ouhbi, Sofia; Isomursu, Minna	2019	10.2196/14537
	Chen 2016	**A study to determine the most popular lifestyle smartphone applications and willingness of the public to share their personal data for health research**	Chen, Juliana; Bauman, Adrian; Allman-Farinelli, Margaret	2016	10.1089/tmj.2015.0159
	Mangal 2023	**Returning study results to research participants: Data access, format, and sharing preferences.**	Mangal, Sabrina; Nino de Rivera, Stephanie; Choi, Jacky; Reading Turchioe, Meghan; Benda, Natalie; Sharko, Marianne; Myers,	2023	https://dx.doi.org/10.1016/j.ijmedinf.2022.104955

			Annie; Goyal, Parag; Dugdale, Lydia; Masterson Creber, Ruth		
Focuses on data sharing outside of the field of public health or medicine (even if several participants from medicine or health are included)	Winkler 2021	**Validation of a survey for measuring scientists' attitudes toward data reuse**	Winkler, Christa E.; Berenbon, Rebecca Fay	2021	10.1002/asi.24412
	Tenopir 2020	**Data sharing, management, use, and reuse: Practices and perceptions of scientists worldwide**	Tenopir, Carol; Rice, Natalie M.; Allard, Suzie; Baird, Lynn; Borycz, Josh; Christian, Lisa; Grant, Bruce; Olendorf, Robert; Sandusky, Robert J.	2020	https://dx.doi.org/10.1371/journal.pone.0229003
	Tenopir 2015	**Changes in Data Sharing and Data Reuse Practices and Perceptions among Scientists Worldwide**	Tenopir, Carol; Dalton, Elizabeth D.; Allard, Suzie; Frame, Mike; Pjesivac, Ivanka; Birch, Ben; Pollock, Danielle; Dorsett, Kristina	2015	https://dx.doi.org/10.1371/journal.pone.0134826
	Tenopir 2011	**Data sharing by scientists: practices and perceptions**	Tenopir, Carol; Allard, Suzie; Douglass, Kimberly; Aydinoglu, Arsev Umur; Wu, Lei; Read, Eleanor; Manoff, Maribeth; Frame, Mike	2011	https://dx.doi.org/10.1371/journal.pone.0021101
	Laegsgaard 2008	**Psychiatric genetic testing: Attitudes and intentions among future users and providers**	Laegsgaard, Mett Marri; Mors, Ole	2008	10.1002/ajmg.b.30609
	Bezuidenhout 2018	**Hidden concerns of sharing research data by low/middle-income country scientists**	Bezuidenhout, Louise; Chakauya, Ereck	2018	https://dx.doi.org/10.1080/11287462.2018.1441780
	Casey 2016	**Interorganizational collaboration in public health data sharing**	Casey, C.; Li, J.; Berry, M.	2016	http://dx.doi.org/10.1108/JHOM-05-2015-0082
Focuses on sharing personal health records	Petrova 2017	**Between "the best way to deliver patient care" and "chaos and low clinical value": General Practitioners' and Practice Managers' views on data sharing**	Petrova, Mila; Barclay, Matthew; Barclay, Sam S.; Barclay, Stephen I. G.	2017	https://dx.doi.org/10.1016/j.ijmedinf.2017.05.009

	Powell 2021	**Health Data Sharing in US Nursing Homes: A Mixed Methods Study**	Powell, Kimberly R.; Deroche, Chelsea B.; Alexander, Gregory L.	2021	https://dx.doi.org/10.1016/j.jamda.2020.02.009
Focuses on topics not relevant to KAP related to data sharing	Yoon 2020	**The role of data-reuse experience in biological scientists' data sharing: an empirical analysis**	Yoon, Ayoung; Kim, Youngseek	2020	10.1108/EL-06-2019-0146
	Ross 2016	**Data sharing through an NIH central database repository: a cross-sectional survey of BioLINCC users**	Ross, Joseph S.; Ritchie, Jessica D.; Finn, Emily; Desai, Nihar R.; Lehman, Richard L.; Krumholz, Harlan M.; Gross, Cary P.	2016	https://dx.doi.org/10.1136/bmjopen-2016-012769
	Prince 2018	**Barriers to the secondary use of data in critical care**	Prince, Karl; Jones, Matthew; Blackwell, Alan; Simpson, Alexander; Meakins, Sallyanne; Vuylsteke, Alain	2018	https://dx.doi.org/10.1177/1751143717741082
	Pratap 2019	**Contemporary Views of Research Participant Willingness to Participate and Share Digital Data in Biomedical Research**	Pratap, Abhishek; Allred, Ryan; Duffy, Jaden; Rivera, Donovan; Lee, Heather Sophia; Renn, Brenna N.; Arean, Patricia A.	2019	https://dx.doi.org/10.1001/jamanetworkopen.2019.15717
	Polanin 2019	**A data-sharing agreement helps to increase researchers' willingness to share primary data: results from a randomized controlled trial**	Polanin, Joshua R.; Terzian, Mary	2019	https://dx.doi.org/10.1016/j.jclinepi.2018.10.006
	Patil 2016	**Public preferences for electronic health data storage, access, and sharing - evidence from a pan-European survey**	Patil, Sunil; Lu, Hui; Saunders, Catherine L.; Potoglou, Dimitris; Robinson, Neil	2016	https://dx.doi.org/10.1093/jamia/ocw012
	Karasneh 2019	**Patient Data Sharing and Confidentiality Practices of Researchers in Jordan**	Karasneh, Reema A.; Al-Azzam, Sayer I.; Alzoubi, Karem H.; Hawamdeh, Sahar S.; Muflih, Suhaib M.	2019	https://dx.doi.org/10.2147/RMHP.S227759
	Karasneh 2021	**Physicians' Knowledge, Perceptions, and Attitudes Related to Patient Confidentiality and Data Sharing**	Karasneh, Reema; Al-Mistarehi, Abdel-Hameed; Al-Azzam, Sayer; Abuhammad, Sawsan; Muflih, Suhaib M.; Hawamdeh, Sahar; Alzoubi, Karem H.	2021	https://dx.doi.org/10.2147/IJGM.S301800

Natsiavas 2019	**Citizen Perspectives on Cross-Border eHealth Data Exchange: A European Survey...MEDINFO 2019, the 17th World Congress on Medical and Health Informatics, August 25-30, 2019, Lyon, France**	Natsiavas, Pantelis; Kakalou, Christine; Votis, Kostas; Tzovaras, Dimitrios; Koutkias, Vassilis	2019	10.3233/SHTI190317
Middleton 2020	**Global Public Perceptions of Genomic Data Sharing: What Shapes the Willingness to Donate DNA and Health Data?**	Middleton, Anna; Milne, Richard; Almarri, Mohamed A.; Anwer, Shamim; Atutornu, Jerome; Baranova, Elena E.; Bevan, Paul; Cerezo, Maria; Cong, Yali; Critchley, Christine; Fernow, Josepine; Goodhand, Peter; Hasan, Qurratulain; Hibino, Aiko; Houeland, Gry; Howard, Heidi C.; Hussain, S. Zakir; Malmgren, Charlotta Ingvoldstad; Izhevskaya, Vera L.; Jedrzejak, Aleksandra; Jinhong, Cao; Kimura, Megumi; Kleiderman, Erika; Leach, Brandi; Liu, Keying; Mascalzoni, Deborah; Mendes, Alvaro; Minari, Jusaku; Wang, Nan; Nicol, Dianne; Niemiec, Emilia; Patch, Christine; Pollard, Jack; Prainsack, Barbara; Riviere, Marie; Robarts, Lauren; Roberts, Jonathan; Romano, Virginia; Sheerah, Haytham A.; Smith, James; Soulier, Alexandra; Steed, Claire; Stefansdottir, Vigdis; Tandre, Cornelia; Thorogood, Adrian; Voigt, Torsten H.; West, Anne V.; Yoshizawa, Go; Morley, Katherine I.	2020	https://dx.doi.org/10.1016/j.ajhg.2020.08.023
Joukes 2015	**End-User Experiences and Expectations Regarding Data Registration and Reuse Before the Implementation of a (New) Electronic Health Record: A Case Study in Two University Hospitals**	Joukes, E.; de Keizer, N.; Abu-Hanna, A.; de Bruijne, M.; Cornet, R.	2015	
Lemke 2011	**Broad data sharing in genetic research: views of institutional review board professionals**	Lemke, Amy A.; Smith, Maureen E.; Wolf, Wendy A.; Trinidad, Susan Brown; Grrip Consortium; Boughman J, Burke W. Dressier L. Edwards K. Freeman W. Gerson N. Juengst E. Lewis S. Marshall P. O'Rourke P. Ponsaran R. Press N. Griffin M. Q. Starks H. Wiesner G.	2011	

Krahe 2020	**Developing a strategy to improve data sharing in health research: A mixed-methods study to identify barriers and facilitators**	Krahe, Michelle A.; Wolski, Malcolm; Mickan, Sharon; Toohey, Julie; Scuffham, Paul; Reilly, Sheena	2020	https://dx.doi.org/10.1177/1833358320917207
Campbell 2003	**Data-sharing and data-withholding in genetics and the life sciences: results of a national survey of technology transfer officers**	Campbell, Eric G.; Bendavid, Eran	2003	
Berry 2012	**Public perspectives on consent for the linkage of data to evaluate vaccine safety**	Berry, Jesia G.; Gold, Michael S.; Ryan, Philip; Duszynski, Katherine M.; Braunack-Mayer, Annette J.; Vaccine Assessment Using Linked Data Working, Group; Gold Ms, Braunack-Mayer A. J. Ryan P. Duszynski K. M. Berry J. G. Xafis V. Carlson J. Richards B. Street J. M. Taylor L. K. Buttery J. P. Freemantle C. J. McNeil J. J. Demos L. L. Thomson C. Lawrence G. L. Elliot E. J. Leask J. Givney R. Lacey G. Woollacott T. Gascoigne C. Koehler A. Watson M. Horgan R. Lawson S. Richmond P. C. Tarrant S. Roughead E. E.	2012	https://dx.doi.org/10.1016/j.vaccine.2012.04.056
Ballard 2020	**Exploring broad consent in the context of the 100,000 Genomes Project: a mixed methods study**	Ballard, Lisa M.; Horton, Rachel H.; Dheensa, Sandi; Fenwick, Angela; Lucassen, Anneke M.	2020	https://dx.doi.org/10.1038/s41431-019-0570-7
AlBaghal 2016	**Obtaining data linkage consent for children: Factors influencing outcomes and potential biases**	Al Baghal, Tarek	2016	10.1080/13645579.2015.1064635
Despotou 2020	**Evaluation of patient perception towards dynamic health data sharing using blockchain based digital consent with the Dovetail digital consent application: A cross sectional exploratory study**	Despotou, George; Evans, Jill; Nash, William; Eavis, Alexandra; Robbins, Tim; Arvanitis, Theodoros N.	2020	https://dx.doi.org/10.1177/2055207620924949
deVet 2003	**Access to data from European registries for epidemiological research: results from a survey by the International Epidemiological Association European Federation**	de Vet, Henrica C. W.; Dekker, Jacqueline M.; Van Veen, Evert Ben; Olsen, Jorn	2003	

	Curran 2014	**A national survey of immunization programs regarding immunization information systems data sharing and use**	Curran, Eileen A.; Seib, Katherine G.; Wells, Katelyn; Hannan, Claire; Bednarczyk, Robert A.; Hinman, Alan R.; Omer, Saad B.	2014	https://dx.doi.org/10.1097/PHH.0000000000000023
	Abuhammad 2020	**Knowledge and Practice of Patients' Data Sharing and Confidentiality Among Nurses in Jordan**	Abuhammad, Sawsan; Alzoubi, Karem H.; Al-Azzam, Sayer I.; Karasneh, Reema A.	2020	https://dx.doi.org/10.2147/JMDH.S269511
	Abele-Brehm 2019	**Attitudes toward Open Science and public data sharing: A survey among members of the German Psychological Society**	Abele-Brehm, Andrea E.; Gollwitzer, Mario; Steinberg, Ulf; Schönbrodt, Felix D.	2019	10.1027/1864-9335/a000384
	Roschka 2022	**Secondary use of health care data and left-over biosamples within the 'Medical Informatics Initiative' (MII): a quasi-randomized controlled evaluation of patient perceptions and preferences regarding the consent process.**	Roschka, Sybille; Leddig, Torsten; Bullerjahn, Mandy; Richter, Gesine; Liedtke, Wenke; Langanke, Martin; Hoffmann, Wolfgang	2022	https://dx.doi.org/10.1186/s12911-022-01922-6
Not a peer reviewed article	Lamas 2018	**Patients' Perception of Privacy of Personal Data, Shared in Online Communities: Are We in the Presence of a Paradox?**	Lamas, Eugenia; Coquedano, Carla; Bousquet, Cedric; Ferrer, Marcela; Chekroun, Michael; Zorrilla, Sergio; Salinas, Rodrigo	2018	
	Ghafur 2020	**Public perceptions on data sharing: key insights from the UK and the USA**	Ghafur, Saira; Van Dael, Jackie; Leis, Melanie; Darzi, Ara; Sheikh, Aziz	2020	https://dx.doi.org/10.1016/S2589-7500(20)30161-8
		**Purpose Outweighs Consent for Health Data Sharing, Consumers Say**		2015	
