# Supplementary material for: How do we measure data sharing in the biomedical sciences? A measurement systematic review of biomedical data sharing-related knowledge, attitudes and practices across stakeholder groups, data types and geographies
Source: BMJ Open. 2026 Mar 11;16(3):e100314. doi: 10.1136/bmjopen-2025-100314 (PMC12983744; doi:10.1136/bmjopen-2025-100314)
Supplement: online supplemental file 4 [file bmjopen-16-3-s004.pdf]

Appendix Table S4. Overview of survey instruments for measuring knowledge, attitudes, and/or practices related to sharing participant-level public health-related data

No	Instrument name	Location (country)	Research objective	Population	Sample size	Data types	Public health field	Full survey available	Instrument domains	Instrument languages	Survey administration	Survey development	Recall period	Measurement properties
1	Patient and public views on data sharing for AI research (Aggarwal, 2021)[30]	England	To obtain views on data sharing (specifically for AI research purposes) and views on using AI on health care data.	Patients (outpatient waiting areas and inpatient wards)	408	EMR, imaging	General	Yes	- Demographics (7)<br>- Health data usage for research (3)<br>- Views AI & ML (7)<br>- Views on data sharing, consent and anonymization (4)	English	1x paper-based survey	Literature review, workshop involving patients and subject matter experts, pilot testing	Future preferences	Reported
2	Attitudes of researchers toward sharing biomedical data (Al-Ebbini 2020)[33]	Jordan	To explore attitudes of biomedical researchers toward data sharing.	Biomedical researchers	195	Clin-epi, genetic	Biomedical research	Yes	- Demographics (6)<br>- Sharing own research data (4)	English	1x, survey administration is NS	Literature review	Mixed	NR
3	KAP of the general public towards the Saudi Human Genome Program (SHGP)	Kingdom of Saudi Arabia	To understand the general public's KAP towards the SHGP, sharing	General public	804	Genetic	General	Yes	- Demographics (5)<br>- Awareness of genetic diseases (6)<br>- Awareness of the SHGP (8)	NR	1x web-based survey	NR	Mixed	NR

No	Instrument name	Location (country)	Research objective	Population	Sample size	Data types	Public health field	Full survey available	Instrument domains	Instrument languages	Survey administration	Survey development	Recall period	Measurement properties
	(Alrefaei, 2022)[32]		genetic data from the SHGP, and the role of AI in genetic data analysis.						- Knowledge and attitudes toward genetic data privacy of the SHGP (9)<br>- Attitudes toward the use of AI in genomics and privacy management of genetic data (6)<br>- Attitude toward sharing genetic data for scientific research (2)					
4	Willingness to share eHealth data for family medicine research (Bartlett 2018)[71]	Canada	To determine the attitudes of family medicine patients toward sharing their eHealth data for research.	Family medicine patients and acute care patients at a respiratory clinic	474	EMR	General	Yes	- Demographics (7)<br>- Scenario-based survey with 3 research scenarios*	French and English	1x paper-based survey	NR	NA	NR

No	Instrument name	Location (country)	Research objective	Population	Sample size	Data types	Public health field	Full survey available	Instrument domains	Instrument languages	Survey administration	Survey development	Recall period	Measurement properties
5	Willingness to share genetic data for science (Bearth 2020)[34]	Switzerland	To offer insights into people's knowledge, perceptions and preferences regarding sharing their genetic data.	Consumer panel members	416	Genetic	General	No	- Demographics (8)<br>- WTS genetic data (3)<br>- Risk perception (5)<br>- Benefit perception (5)<br>- Trust (3)<br>- Knowledge about the use of genetic data and privacy of data sharing (7)	German	1x web-based survey	NR	Future preferences	NR
6	Preferences of sharing medical health record data (Bell 2014)[92]	United States of America	To establish a baseline on patient preferences regarding sharing their medical health records.	General public	70	EMR	General	Yes	- Demographics (8)<br>- What am I sharing? (sensitive vs. non-sensitive information) (9)<br>- Who am I sharing with? (2)<br>- Which type of funding do I allow? (4)	English	1x web-based survey	NR	Future preferences	NR
7	Attitudes toward secondary use of general practice	Australia	To understand community views on	General public	2,604	Clin-epi	General	Yes	- Eligibility criteria (3)<br>- Demographics (6)	English	1x web-based survey	Review of pre-existing surveys,	Mixed	NR

No	Instrument name	Location (country)	Research objective	Population	Sample size	Data types	Public health field	Full survey available	Instrument domains	Instrument languages	Survey administration	Survey development	Recall period	Measurement properties
	data (Braunack-Mayer, 2024)[81]		sharing general practice data for secondary purposes.						- Patient information in general practice (2)<br>- For what reasons can general practice information be shared? (2)<br>- Sharing and linking general practice information for research (1)<br>- Trust in GP and general practice records (2)			literature review, FGD		
8	Data Sharing in ENGAGE (Budin-Ljøsne, 2014)[22]	Europe	To understand the data-sharing experiences of ENGAGE consortium members.	Researchers	26	Clin-epi, genetic	General	No	- Barriers and facilitators of data sharing that apply to collaborations with/ within ENGAGE (13: 10 multiple choice, 3 open-ended)	English	1x web-based survey	NR	Mixed	NR

No	Instrument name	Location (country)	Research objective	Population	Sample size	Data types	Public health field	Full survey available	Instrument domains	Instrument languages	Survey administration	Survey development	Recall period	Measurement properties
9	Data sharing of imaging trials in radiology (Bosserdt 2019)[68]	Europe	To determine future directions of clinical trials and data sharing in radiology.	Researchers †	460	Imaging	General	Yes	- General (about participants' affiliations, etc.) (3)<br>- Own randomised imaging trials (15)<br>- Own publications on randomised imaging trials (3)<br>- Own data sharing on randomised imaging trials (2)<br>- Involvement in third-party publications on randomised imaging trials (2)<br>- Sharing data from imaging trials (6)<br>- Access to data from imaging trials of other groups (8)<br>- Own interests regarding conducting imaging trials, sharing or receiving data (5)	English	1x web-based survey	NR	Mixed	NR

No	Instrument name	Location (country)	Research objective	Population	Sample size	Data types	Public health field	Full survey available	Instrument domains	Instrument languages	Survey administration	Survey development	Recall period	Measurement properties
10	Public preferences for digital health data sharing (Biasiotto, 2023) (Johansson 2021)[41,82,88] 11/03/2026 11:47:00	Initially conducted in Sweden, Norway, Iceland, and the United Kingdom. Extended to Austria, Denmark, France, Germany, Ireland, Italy, the Netherlands, and Spain.	To investigate the general public's preferences for sharing digital health data.	General public	1,967 (initial survey) and 5,015 (extended survey in 8 countries)	Clin-epi, EMR	General	No	- Demographics (NR)<br>- DCE with five attributes: data collector, data user, reasons for data use, information on data sharing and consent, availability of review process<br>- 18 levels in total: three or four per attribute<br>- Attitudes and trust (NR)	English and the respective national language of each country	1 x web-based survey	Literature review, FGDs, nominal group technique, webinar with content experts, think-aloud interviews, cognitive interviews, workshop involving subject matter experts, online ranking survey, pilot testing	NA	Reported
11	Attitudes of genomic research participants towards data sharing (Burstein 2014, McGuire 2011, Oliver 2012)[52,86,87]	United States of America	To explore the attitudes of parents of paediatric genomic research participants and adult research	Genomic research participants and parents of paediatric research participants	309	Clin-epi, genetic	(Pediatric) Conditions and diseases‡	No	- Demographics (7)<br>- Understanding of participation in research generally and genetic information (NR)<br>- Comfort in decision-making (NR)	English	1x survey administered using QDS, and participants were guided through	Literature review, subject matter expert input	Future preferences	NR

No	Instrument name	Location (country)	Research objective	Population	Sample size	Data types	Public health field	Full survey available	Instrument domains	Instrument languages	Survey administration	Survey development	Recall period	Measurement properties
			participants toward data sharing.						- Trust in medical researchers (1)<br>- Risk-benefit assessment (2)<br>- Preferences for and attitudes toward consent types and data-sharing options (NR)		the survey in person			
12	Public comprehension of health data privacy protections (Corman, 2022)[93]	Australia	To assess how accurately people understood the effectiveness of techniques for protecting the privacy of shared health data.	The general public (most of them were university students)	317	EMR	General	Yes	- Demographics and payment (12)<br>- Understanding and opinions of four data-sharing methods:<br>- Complete EHR record (9)<br>- Deidentified EHR data (10)<br>- Aggregate data (9)<br>- Aggregate data using differential privacy (9)	English	1x web-based survey	NR	Mixed	NR

No	Instrument name	Location (country)	Research objective	Population	Sample size	Data types	Public health field	Full survey available	Instrument domains	Instrument languages	Survey administration	Survey development	Recall period	Measurement properties
									- Privacy attitudes (15)					
13	Rare disease patients' perspectives on data sharing and data protection in research and healthcare settings (Courbier 2019)[46]	Europe (a small percentage of other countries outside Europe)	To explore patient and family perspectives on data sharing and data protection in research and healthcare settings and develop recommendations to support the shaping of future data-sharing initiatives in rare disease research.	Patients with rare diseases and family members	2,013	Clin-epi, genetic	Rare diseases	Yes	- Demographics (7)<br>- Sensitivity of health information and perceived risks of sharing data (7)<br>- Trust placed in stakeholders involved in data sharing (10)<br>- WTS data for different purposes (6)<br>- Information needed/ wanted to be willing to share data (5)<br>- Control needed/ wanted over health data (3)	Translated into 23 languages	1x web-based survey	Review of pre-existing surveys, subject matter expert input, FGDs, Delphi exercises, group discussions with patient advisory council	Future preferences	NR

No	Instrument name	Location (country)	Research objective	Population	Sample size	Data types	Public health field	Full survey available	Instrument domains	Instrument languages	Survey administration	Survey development	Recall period	Measurement properties
14	Attitudes toward information and consent for the secondary use of health data (Cumyn 2021)[58]	Canada	To assess citizens' opinions, REC members and researchers regarding information and consent for the secondary use of health data for research within learning health systems.	The general public, researchers, and REC members	387 citizens, 66 researchers, 99 REC members	EMR	General	Yes	- Demographics (6)<br>- Secondary use of health data (1)<br>- Level of information and consent needed for the use of de-identified health data (6)<br>- Acceptability of a proposed meta-consent model (3)<br>- Delegation of consent to a third party (2)<br>- Individual control versus societal benefits of research (1)	French and English	1x, over the phone for citizens, web-based for researchers and REC members	Literature review, subject matter expert input, survey pretesting	Future preferences	NR
15	Data sharing in rare diseases (Darquy 2016)[61]	France, Germany, Italy, Belgium, Spain	To explore patients' and family members' views on medical data sharing when	LDs patients and their close relatives	195	NR	Leukodystrophies (rare diseases)	Yes	- Demographics (5)<br>- Views on personal health data sharing (11)<br>- Views on family genetic data sharing (4)	French, English, Spanish, Italian, and German	1x web-based survey	Subject matter expert input	Mixed	NR

No	Instrument name	Location (country)	Research objective	Population	Sample size	Data types	Public health field	Full survey available	Instrument domains	Instrument languages	Survey administration	Survey development	Recall period	Measurement properties
			compiling a European LDs database.						- Willingness to participate in the proposed database (6)<br>- Database as a bridge to clinical trials (8)					
16	Public attitudes towards genomic data sharing (Etchegary, 2023 Etchegary, 2021)[65,94,95]	Canada	To understand public attitudes towards genomic data sharing.	General public	697	Genetic	General	Yes	Vignettes followed by multiple choice/open-ended questions on:<br>- Genetic testing (4)<br>- Patient consent (2)<br>- Incidental findings (4)<br>- Information you would want (5)<br>- Concerns (1)<br>- Use of data (3)<br>- Secondary use of data (3)<br>- General information (7)<br>- Drug reactions (1)	English	1x web-based survey	Literature review, subject matter expert input	Mixed	NR

No	Instrument name	Location (country)	Research objective	Population	Sample size	Data types	Public health field	Full survey available	Instrument domains	Instrument languages	Survey administration	Survey development	Recall period	Measurement properties
									- Family history (4)<br>- Final thoughts (1)					
17	Attitudes of clinical and scientific research staff toward biomedical data sharing (Federer 2015)[70]	United States of America	To better understand the motivations and barriers to data sharing among researchers.	Researchers	190	NR	General	Yes	- Demographics (7)<br>- Practices and attitudes toward data sharing (4)<br>- Data management tasks (9)<br>- Data management and sharing practices (4)	English	1x web-based survey	Pilot testing	Experience	NR
18	Participant Issues and Expectations Project (PIP) (Goodman 2017, Goodman 2016)[59,85]	United States of America	To assess views and preferences of research participants on the use of de-identified data in large research datasets.	Research participants, their relatives, and the general public§	450 (228 cancer patients, 155 controls, 67 relatives)	Genetic	Cancer	No	- Demographics (7)<br>22 items across the following domains:<br>- Decision to participate in research<br>- Relationship between researchers and participants	English	1x web-based survey	Cognitive interviews with research participants, subject matter expert input, survey pretesting, pilot testing,	Future preferences	Reported

No	Instrument name	Location (country)	Research objective	Population	Sample size	Data types	Public health field	Full survey available	Instrument domains	Instrument languages	Survey administration	Survey development	Recall period	Measurement properties
									- Re-consent and broad consent<br>- Return of results<br>- Sharing data with different stakeholders<br>- Use and security of de-identified data<br>- Family communication of health issues					
19	Public preferences about secondary uses of electronic health information (Grande 2013)[39]	United States of America	To explore public attitudes toward the secondary use of electronically available personal health information.	General public[¶]	3,336	EMR	General	No	- Demographics (7)<br>- Health status and healthcare access measures (3)<br>- Scenarios describing secondary data use constructed with 3 attributes: use, users, data sensitivity (genetic information)[#]	NR	1x web-based survey	NR	NA	NR

No	Instrument name	Location (country)	Research objective	Population	Sample size	Data types	Public health field	Full survey available	Instrument domains	Instrument languages	Survey administration	Survey development	Recall period	Measurement properties
									- Health care system mistrust (9)					
20	Factors influencing personal health data sharing (Helou 2021)[60]	Middle East, Asia, Europe (a small percentage of North America, Oceania, and South America)	To examine the relationship between personal health data's perceived usefulness, sensitivity, and anonymity and people's willingness to share with researchers.	Healthcare workers, researchers, IT workers, and other	112	EMR, mental health records	General	No	- Demographics (5) 31 items across the following domains: - Willingness to share data - Perception of sensitivity of data - Perception of usefulness of data - Perception of anonymity of data	English	1x web-based survey	Literature review, survey pretesting	Future preferences	NR
21	Attitudes of Australian breast cancer patients toward the secondary use of administrative and clinical trial data	Australia	To understand the attitudes of patients who have been diagnosed with breast cancer toward the secondary use	Individuals diagnosed with breast cancer	132	Clin-epi	Breast cancer	Yes	- Demographics (8) - Support for the secondary use of de-identified health data (9) - Assumptions about current health data use (4)	English	1x web-based survey	Literature review, interviews with subject matter experts	Mixed	NR

No	Instrument name	Location (country)	Research objective	Population	Sample size	Data types	Public health field	Full survey available	Instrument domains	Instrument languages	Survey administration	Survey development	Recall period	Measurement properties
	(Hutchings, 2023)[79]		and sharing of de-identified administrative health and clinical trial data.						- Perceived benefits of data sharing (2)<br>- Perceived barriers to data sharing (2)<br>- Opinions on consent (5)<br>- Data sharing with registries (2)<br>- Linking data (2)<br>- Concerns about privacy (4)<br>- Concerns about information security (2)<br>- Concerns about data sharing (1)<br>- Data ownership (2)<br>- Sharing and public funding (1)<br>- Data sharing preferences (1)					
22	Health data sharing perspectives of patients receiving	United States of America	To understand cancer patients'	Patients (receiving	1,200	EMR	Cancer	Yes	- Health status and demographics (17)	English	1x paper-based survey	Literature review, patient input, review of	Mixed	NR

No	Instrument name	Location (country)	Research objective	Population	Sample size	Data types	Public health field	Full survey available	Instrument domains	Instrument languages	Survey administration	Survey development	Recall period	Measurement properties
	care in CancerLinQ-participating oncology practices (Jagsi 2023)[72]		experiences and perspectives on data sharing.	oncology care)					- Knowledge of nationwide cancer databases (9)<br>- Views and concerns about sharing data for cancer databases (26)<br>- Views on the health care system and doctors (20)<br>- Concerns about privacy (2)			pre-existing surveys, cognitive interviews, subject matter expert input, survey pretesting		
23	Patient perspectives on sharing deidentified trial data (Jones 2016)[80]	United States of America	To investigate patients' attitudes toward sharing deidentified individual-patient data from clinical trials.	Hospital patients	799	Clin-epi	General	Yes	- Attitudes toward participating in research studies (2)<br>- Motivations for participating in a clinical trial (6)<br>- Views on clinical trials in general (12)<br>- Views on results of clinical trials being shared (3)	English	1x survey administration NR	NR	Future preferences	NR

No	Instrument name	Location (country)	Research objective	Population	Sample size	Data types	Public health field	Full survey available	Instrument domains	Instrument languages	Survey administration	Survey development	Recall period	Measurement properties
24	Public opinion on sharing data from health services for clinical and research purposes without explicit consent (Jones 2022)[29]	United Kingdom	To assess the patients' views on sharing mental and physical health data for clinical and research purposes.	General public	29,275	EMR	General	Yes	- Understanding of current NHS data-sharing practices (2)<br>- Personal experience with mental health/physical health conditions (5)<br>- WTS identifiable physical health/mental health data (8)<br>- WTS de-identified structured physical health/mental health data (12)<br>- WTS de-identified, free-text physical health/mental health data (12)<br>- Views on potential systems for managing data	English	1x web-based survey	Research advisory group input, survey pretesting, pilot testing	Mixed	NR

No	Instrument name	Location (country)	Research objective	Population	Sample size	Data types	Public health field	Full survey available	Instrument domains	Instrument languages	Survey administration	Survey development	Recall period	Measurement properties
									consent in the NHS (34)<br>- Demographics (9)					
25	Individual willingness to share personal health information with secondary information users in South Korea (Jung 2020)[24]	South Korea	To investigate individuals' willingness to share their health information under different circumstances.	General public	104	EMR	General	Yes	- WTS with different information users (4)<br>- WTS under different conditions based on anonymity (2)<br>- WTS based on type of information (2)	NR	1x web-based survey	NR	Future preferences	NR
26	Researchers' perspectives on data sharing in LMICs (Kaewkungwal 2020)[25]	Thailand	To examine the views of researchers in Thailand regarding issues and challenges associated with data sharing.	Researchers	229	Clin-epi, genetic, imaging	General	Yes	- Demographics (3)<br>- Views on data sharing (12)<br>- Challenges and barriers in data sharing faced by researchers (9)	English and Thai	1x, both paper- and web-based versions	Literature review	Current views assessed	NR

No	Instrument name	Location (country)	Research objective	Population	Sample size	Data types	Public health field	Full survey available	Instrument domains	Instrument languages	Survey administration	Survey development	Recall period	Measurement properties
27	Biobank participants' attitudes toward data sharing and privacy (Kasperbauer 2022, Kasperbauer 2021, Beskow 2017)[51,89,97]	United States of America	To assess biobank participants' understanding of consent forms, their attitudes toward data sharing, and their trust in the biobank.	Biobank participants	22	Clin-epi	General	Yes	- Knowledge test about previously signed consent form (11)<br>- Satisfaction with informed consent process (5)<br>- Demographics (NR)	English	1x phone-based survey	Review of pre-existing surveys, patient advisory group input	Current experience	NR
28	Patient perspectives on medical and biospecimen data sharing for research (Kim 2019)[73]	United States of America	To investigate the impact of different consent form presentations on EMR and biospecimen sharing rates, considering user interface design, data recipients, and patient characteristics.	Outpatients	1,246	EMR	General	No	- WTS 59 data and biospecimen items (yes/no) with different institutions	English and Spanish	1x, both paper- and web-based versions	FGDs, pilot testing	Future preferences	NR

No	Instrument name	Location (country)	Research objective	Population	Sample size	Data types	Public health field	Full survey available	Instrument domains	Instrument languages	Survey administration	Survey development	Recall period	Measurement properties
29	Views on electronic data sharing for healthcare and research (Kim 2015, Kim 2017)[83,84]	United States of America	To investigate the public's views on data sharing for healthcare and research.	General public	800	EMR	General	Yes	- Demographics (9)<br>- Electronic health record data sharing (8)<br>- Data sharing for research (8)<br>- Health status (3)	English and Spanish	1x phone-based survey	Literature review, review of pre-existing surveys, FGDs, survey pretesting	Future preferences	NR
30	Willingness to share mental health data (Kirkham 2022)[31]	United Kingdom	To investigate factors influencing mental health data sharing for research purposes among people with and without experience of mental illness.	The general public (NHS users)	2,187	EMR	Mental health	Yes	- Views about sharing mental health and physical health data (46)<br>- Personal experiences with mental health and physical health (24)<br>- Demographics (6)	English	1x web-based survey	Patient and subject matter expert input	Future preferences	NR
31	Secondary use of clinical data for biomedical research	Germany	To assess physicians' views and concerns about	Physicians	446	Clin-epi	General	Yes	- Physician activity and previous experience with secondary use of clinical data (10)	German	1x web-based survey	Literature review, interviews with subject matter experts, survey	Mixed	NR

No	Instrument name	Location (country)	Research objective	Population	Sample size	Data types	Public health field	Full survey available	Instrument domains	Instrument languages	Survey administration	Survey development	Recall period	Measurement properties
	(Köngeter 2024)[35]		secondary data use and their willingness to adapt workflows to enable data sharing.						- Views on planned research use of clinical data (6)<br>- Possible adjustments to physician activity (11)			pretesting (cognitive interviews)		
32	Barriers and facilitators to data sharing in health research (Krahe 2023)[75]	Australia	To evaluate health researchers' data management practices and identify barriers and facilitators to data sharing to inform a behaviour change intervention strategy.	Researchers	77	Clin-epi	General	Yes	- Researcher's current activity (6)<br>- Views and concerns about data sharing (10)<br>- Motivation for data sharing (8)<br>- WTS data (8)<br>- Past data-sharing experiences (6)	English	1x web-based survey	NR	Mixed	NR
33	Sharing Data for Precision Medicine	Singapore	To look at priorities and preferences	General Public	1,000	Clin-epi, genetic	Precision medicine	Yes	- WTS data (2)<br>- Trust in institutions (13)	English, Mandarin, Malay	1x using a survey platform	FGDs, review of pre-existing surveys, pilot	Future preferences	NR

No	Instrument name	Location (country)	Research objective	Population	Sample size	Data types	Public health field	Full survey available	Instrument domains	Instrument languages	Survey administration	Survey development	Recall period	Measurement properties
	(Lysaght Tamra 2021)[26]		for sharing de-identified data for precision medicine.						- Trade-offs in data sharing agreements (scenario-based)<br>- Demographics (8)			testing		
34	Minority youth's perspectives on data sharing (Matson 2019)[50]	United States of America	To assess sexual and gender minority youth's perspectives on data sharing and their trust in researchers.	Sexual and gender minority youth	197	Clin-epi	Sexual health and HIV	Yes	- Attitudes toward sexual health survey (3)<br>- Trust in researchers (6)<br>- Views on data sharing (8)<br>- Demographics (6)	English	1x web-based survey	NR	Future preferences	NR
35	Survey on Sharing Data From Clinical Trials (Mello 2018)[53]	United States of America	To determine clinical trial participants' perceptions of sharing data from clinical trials.	Clinical trial participants or their parents/guardians	771	Clin-epi	Nutrition, diabetes, tobacco use, kidney disease, cardiovascular disease, cancer	Yes	- General questions about participation in clinical trials (6)<br>- Opinions on data sharing (9)<br>- Advice on seeking permission for data sharing (5)<br>- Data sharing systems (3)	English	1x paper-based survey	FGDs, input from subject matter experts, pilot testing (cognitive interviews)	Mixed	NR

No	Instrument name	Location (country)	Research objective	Population	Sample size	Data types	Public health field	Full survey available	Instrument domains	Instrument languages	Survey administration	Survey development	Recall period	Measurement properties
									- Personal details (7)					
36	Attitudes Toward Sharing Genomic Data (Middleton 2014)[40]	International	To ascertain attitudes towards sharing genomic data.	The general public, genetic health professionals, other health professionals, genomic researchers	6,944	Genetic	General	No	32 items across the following domains:<br>- Questions about you<br>- Sharing of pertinent findings<br>- Sharing of incidental findings<br>- Categorizing incidental findings<br>- Relations with risk<br>- Raw data<br>- Duty of genomic researchers<br>- Filter of genomic information<br>- Consent for genomic research	English	1x web-based survey	Literature review, FGDs, survey pretesting, pilot testing	Future preferences	Reported
37	Your DNA, Your Say (Milne, 2019 and 2021)[47,48]	22 countries	To examine public perspectives	General public	36,268	Genetic	General	Yes	- Online footprint (7)	16 languages	1x web-based survey	Literature review, survey pretesting, pilot testing	Future preferences	Reported

No	Instrument name	Location (country)	Research objective	Population	Sample size	Data types	Public health field	Full survey available	Instrument domains	Instrument languages	Survey administration	Survey development	Recall period	Measurement properties
			on genomic data sharing.						- Medical data access by others (9)<br>- Perceived harms of sharing medical and DNA information (17)<br>- Expectations of information (4)<br>- Trust (11)<br>- Demographics (8)					
38	Attitudes Toward Data Collection, Ownership, and Sharing Amongst Those with Parkinson's Disease (Mursaleen 2017)[49]	20 countries, with the majority of the responses from the United Kingdom, the United States of America and Canada	To determine attitudes towards data collection, ownership, and sharing among patients with Parkinson's Disease.	Patients with Parkinson's Disease	394	Clin-epi	Parkinson's Disease	Yes	- Background information (7)<br>- Health confidence (11)<br>- Sharing data (17)<br>- Future correspondence (2)	English	1x web-based survey	Patient advisory group input	Mixed	NR

No	Instrument name	Location (country)	Research objective	Population	Sample size	Data types	Public health field	Full survey available	Instrument domains	Instrument languages	Survey administration	Survey development	Recall period	Measurement properties
39	Pharmaceutical Companies' Secondary Uses of Patient Records (Nakada 2020)[27]	Japan	To assess public attitudes toward pharmaceutical companies' secondary use of EMR data and preferences regarding different types of consent.	General public	3,000	EMR	General	Yes	- Awareness of "clinical trials" (2)<br>- Awareness of the processes of drug development, such as cost, time, and the number of candidate substances in a new drug (3)<br>- Knowledge of the laws and regulations for use of patient records in Japan (4)<br>- Assessment of the public benefit of the secondary use of patient records (10)<br>- Preferences for consent for the secondary use of patient records (6)<br>- Basic characteristics of	Japanese	1x web-based survey	NR	Mixed	NR

No	Instrument name	Location (country)	Research objective	Population	Sample size	Data types	Public health field	Full survey available	Instrument domains	Instrument languages	Survey administration	Survey development	Recall period	Measurement properties
									the respondents (7)					
40	International Survey on Data Sharing and Re-use in Traumatic Stress Research (Prakash 2023)[69]	Australia, Brazil, Chile, France, Japan, Lebanon, Netherlands, South Korea, and the United States of America	To understand traumatic stress researchers' perspectives and practices regarding FAIR data, including identifying barriers and creating a toolkit for FAIR data practices.	Traumatic stress researchers	222	EMR, genetic	Traumatic stress	Yes	- Demographics (5)<br>- Perceived concerns about data re-use (6)<br>- Perceived effort required for data re-use (18)<br>- Ethical considerations as barriers to data sharing (26)<br>- Other constructs related to data sharing and re-use (10)	English, Japanese, Spanish, French, Brazilian Portuguese, Korean, and Arabic	1x web-based survey	Review of pre-existing surveys	Mixed	NR
41	Clinical Trial Data Sharing (Rathi, 2014 and 2012)[76,77]	International	To investigate clinical trialists' opinions and experiences of sharing clinical trial data	Clinical trialists	317	Clin-epi	General	Yes	- Attitudes towards data sharing (10)<br>- Data sharing practices (9)<br>- Concerns about data sharing (3)	English	1x phone or web-based survey	Literature review, subject matter expert input	Mixed	NR

No	Instrument name	Location (country)	Research objective	Population	Sample size	Data types	Public health field	Full survey available	Instrument domains	Instrument languages	Survey administration	Survey development	Recall period	Measurement properties
			outside their research team.						- Experiences with requesting data (5)<br>- Respondent characteristics (11)					
42	Personal Attitudes of Participants Towards Medical Research and Data Donation (Survey 1) (Richter, 2021)[36]	Germany	To determine the personal attitudes of patients towards medical research and data donation.	Hospital outpatients	376	EMR	General	Yes	- Demographics (2)<br>- Understanding and personal views of medical research (12)<br>- Acceptance of data donation (2)<br>- Possible implementations of data donation (5)<br>- If SARS-CoV-2 affected attitudes towards data donation (2)	German	1x paper-based survey	NR	Current views on data sharing	NR
43	Personal Attitudes of Participants Towards Medical Research and Data Donation (Survey 2) (Richter, 2021)[36]	Germany	To verify the results of Survey 1 and to address more specific aspects of a	Hospital outpatients	132	EMR	General	Yes	- Attitudes towards legally permitting research use of pseudonymised medical data	German	1x paper-based survey	NR	Current views on data sharing	NR

No	Instrument name	Location (country)	Research objective	Population	Sample size	Data types	Public health field	Full survey available	Instrument domains	Instrument languages	Survey administration	Survey development	Recall period	Measurement properties
			potential practical implementation of data donation.						without prior consent (1)<br>- Agreement of a general civic duty to improve medical research (1)<br>- Implementation of data donation (2)<br>- Demographics (2)					
44	Sharing Your Data in the Hospital (Richter, 2021)[37]	Netherlands	To determine patient preferences regarding using personal data and specimens for research and broad consent for the future use of their data and specimens.	Patients	5,258	EMR	General	Yes	- Experiences with data sharing for research (3)<br>- Willingness to give consent for research (4)<br>- Reasons for giving or withholding consent (4)<br>- Data reuse knowledge (2)<br>- Willingness to give consent for commercial use (4)<br>- Opinions on and understanding of	Dutch	1x web-based survey	Review of pre-existing surveys	Mixed	NR

No	Instrument name	Location (country)	Research objective	Population	Sample size	Data types	Public health field	Full survey available	Instrument domains	Instrument languages	Survey administration	Survey development	Recall period	Measurement properties
									leaflet containing information about data sharing and reuse (7)<br>- Return of findings (2)<br>- Willingness to give consent post leaflet (7)<br>- Demographics (12)					
45	"Data donation" for Medical Research (Richter, 2021)[37]	Germany	To evaluate attitudes toward medical data donation for research purposes.	General public	1,006	EMR	General	Yes	5 items on WTS in different circumstances, and reason for unwillingness if applicable	German	1x phone-based survey	Review of pre-existing surveys	Future preferences	NR
46	Canadians' opinions on COVID-19 data sharing (SavicKallesoe, 2023)[62]	Canada	To examine Canadians' opinions on public health authorities sharing de-	General public	4,981	Clin-epi, genetic	COVID-19	Yes	- Demographics (5)<br>- Your experience with COVID-19 (4)<br>- Data sharing options (18)<br>- Data of the virus (2)	English and French	1x web-based survey	Literature review, subject matter expert input, pilot testing	Mixed	NR

No	Instrument name	Location (country)	Research objective	Population	Sample size	Data types	Public health field	Full survey available	Instrument domains	Instrument languages	Survey administration	Survey development	Recall period	Measurement properties
			identified COVID-19 data.						- Data of the person (1)<br>- Linking person and virus data (1)<br>- Final comments (1)					
47	Motivations for Data Sharing (Shah, 2019 Shah, 2019)[54,55]	Denmark, Sweden, Netherlands, United Kingdom	To investigate the DIRECT research participants' preferences for control over sharing different types of de-identified data and with whom data can be shared once the project ends.	Research participants	855	Clin-epi, EMR, genetic	Diabetes	Yes	- Section 1: Taking part in DIRECT (6)<br>- Section 2: Sharing DIRECT data (10)<br>- Section 3: About you (8)	English (other languages NR)	1x paper-based survey	Patient and subject matter expert input	Future preferences	NR
48	Determinants of Health Data Sharing Behavior (Silber 2023)[38]	Germany	Determining public trust in science and willingness to	General public	746	EMR	Cancer Research	Yes	- Type of Information (5)<br>-Purpose Information (5)	German	1x web-based survey	Literature review, review of pre-existing survey	Mixed	NR

No	Instrument name	Location (country)	Research objective	Population	Sample size	Data types	Public health field	Full survey available	Instrument domains	Instrument languages	Survey administration	Survey development	Recall period	Measurement properties
			share cancer research data, including understanding the effects of question order, wording, and incentives on consent to data linkage.						- Governance (5)<br>- Anticipated Benefit (5)					
49	Experiences of Sharing GWAS Data with dbGaP (Simpson 2014)[63]	International, with the majority of responses being from the United States of America and Canada	To understand the practical barriers and perceived ethical challenges experienced with sharing GWAS data with dbGaP.	Researchers	175	Clin-epi, genetic	General	No	44 items covering the following domains:<br>- Demographics<br>- Past application for research funding<br>- Contribution of data to dbGaP<br>- Access of data through dbGaP and other similar databases<br>- Avoidance of the data-sharing policy	English	1x web-based survey	Pilot testing	Mixed	NR

No	Instrument name	Location (country)	Research objective	Population	Sample size	Data types	Public health field	Full survey available	Instrument domains	Instrument languages	Survey administration	Survey development	Recall period	Measurement properties
50	Perceptions and Attitudes Toward Data Sharing Among Dental Researchers (Spallek, 2019)[45]	International (part of IADR)	To explore established dental researchers' opinions and past experiences concerning data sharing and management.	Dental health researchers	42	Clin-epi, EMR	Dental health	Yes	- General attitude toward data sharing (2)<br>- Perceptions of sharing data through data repositories (20)<br>- Own experience with sharing data in response to personal requests (38)<br>- Own experience with requesting and obtaining data produced by other investigators (16)<br>- Knowledge and experience regarding data management (14)<br>- Demographics (11)	English	1x web-based survey	Pilot testing	Mixed	NR

No	Instrument name	Location (country)	Research objective	Population	Sample size	Data types	Public health field	Full survey available	Instrument domains	Instrument languages	Survey administration	Survey development	Recall period	Measurement properties
51	Perceptions and Preferences About Data Sharing and Privacy among Behavioral Health Patients (Soni 2019)[44]	United States of America	To understand the perceptions of behavioural health patients regarding data sensitivity, willingness to share health data for care and research and related motivations.	Patients	86	Clin-epi	Behavioural health	Yes	- Demographics (5)<br>- Well-being and mental health status (40)<br>- Data sharing choices for care (33)<br>- Data sharing choices for research (10)	English and Spanish	1x web-based or paper-based	Review of pre-existing surveys, survey pretesting	Future preferences	NR
52	Ethical Concerns on Sharing Genomic Data Including Patients' Family Members (Takashima 2018)[28]	Japan	To understand the attitudes of healthy adults and patients toward sharing clinical and genomic data, including family members.	General public	10,881	Clin-epi, genetic	General	No	NR	Japanese	1x web-based survey	NR	Future preferences	NR

No	Instrument name	Location (country)	Research objective	Population	Sample size	Data types	Public health field	Full survey available	Instrument domains	Instrument languages	Survey administration	Survey development	Recall period	Measurement properties
53	Individual Participant Data Survey (Tan, 2021)[78]	Australia and New Zealand	To survey researchers on their attitudes, motivations, and barriers to sharing IPD.	PIs of interventional trials registered on ANZCTR	281	Clin-epi	General	Yes	- Attitudes toward data sharing (3)<br>- Perceived barriers to data sharing (11)<br>- Motivations for/ against data sharing (2)<br>- Practices and requirements around data sharing (4)<br>- Demographics (2)	English	1x web-based survey	Review of pre-existing surveys, pilot testing	Mixed	NR
54	The relative importance of different attributes in influencing preferences of data sharing<br>Tully, 2020, Aitken 2018[42,43]	Scotland and Sweden	To identify the factors most influential in shaping public preferences for conducting research with linked EMR data.	General public	1,978	EMR	General	No	- Narrative introducing data sharing concepts<br>- Attitudes toward data linkage (5)<br>- DCE with five attributes: who would access linked data, types of data being linked, purpose of research, options for profit making,	English and Swedish	1x web-based survey	Literature review, public involvement panel input, pilot testing (via cognitive interviews)	NA	Reported

No	Instrument name	Location (country)	Research objective	Population	Sample size	Data types	Public health field	Full survey available	Instrument domains	Instrument languages	Survey administration	Survey development	Recall period	Measurement properties
									who would oversee research<br>- Four levels for each attribute<br>- A total of 240 scenario pairs and 20 scenario packs; participants presented with 12 scenario pairs<br>- Demographics (5)					
55	Public preferences for sharing health data in Australia (Varhol 2023)[64]	Australia	To determine public preferences for sharing health data, including understanding the factors influencing individuals' willingness to share their health information.	General Public	1,138	Clin-epi, EMR	General	Yes	- Demographics (9)<br>- Introductory narrative<br>- DCE with five attributes: who would have access to data, type of information, purpose for integration, information governance, anticipated benefit	English	1x web-based survey	Pilot testing	NA	NR

No	Instrument name	Location (country)	Research objective	Population	Sample size	Data types	Public health field	Full survey available	Instrument domains	Instrument languages	Survey administration	Survey development	Recall period	Measurement properties
									- Five levels per attribute on average<br>- 512 possible combinations, participants received 12 each					
56	Barriers and facilitators to sharing health and medical data (Varhol 2022)[56]	Australia	To explore general practitioners' attitudes towards sharing de-identified patient data for secondary research purposes.	General Practitioners	64	Clin-epi, EMR	General	Yes	- Background Information (8)<br>- Public Health Research (3)<br>- Data Sharing & Secondary Use of General Practice Data For Research Purposes (5)<br>- Providing Access To Data For Research (7)<br>- Trust in Data Collection & Storage Organisation (10)<br>- Understanding of Organisations Management and	English	1x web-based survey	NR	Mixed	NR

No	Instrument name	Location (country)	Research objective	Population	Sample size	Data types	Public health field	Full survey available	Instrument domains	Instrument languages	Survey administration	Survey development	Recall period	Measurement properties
									Storage of Data (12)<br>- Concerns Related to Sharing Data for Research (7)					
57	Sharing Genomic Data for Research From Clinical Records (Vidgen, 2020)[66]	Australia	To explore public opinions related to sharing genomic data for research from medical records.	General public	1,494	Genetic (from medical record)	General	Yes	- Demographic questions (7)<br>- Permission and preferences (7)<br>- Your concerns about sharing your genomic data (2)	English	1x web-based survey	Literature review, review of pre-existing surveys	Future preferences	NR
58	Qualitative investigation into responses from the Australian public (Warren 2023)[67]	Australia	To investigate factors affecting public attitudes to data sharing through responses to diverse genomic data sharing scenarios.	General public	243	Genetic	General	Yes	Scenario-based survey with 7 scenarios categorising current Australian genomic data sharing practices in different settings (clinical, research, biobank, data repository); 5 open-ended	English	1x web-based survey	Review of pre-existing survey	NA	NR

No	Instrument name	Location (country)	Research objective	Population	Sample size	Data types	Public health field	Full survey available	Instrument domains	Instrument languages	Survey administration	Survey development	Recall period	Measurement properties
									questions for each scenario indicate WTS.					
59	Sharing Information From Personally Controlled Health Records (Weitzman, 2010)[74]	United States of America	To ascertain attitudes toward sharing information from PCHRs for health research.	Early users of PCHRs	151	PCHR	General	No	NR	English	1x using the PCHR platform	NR	NR	NR
60	Willingness to Share Clinical Data for Research (Weng, 2019)[57]	United States of America	To determine individuals' willingness to share demographic and clinical data to a shared research repository.	Faculty, students, and staff at two major medical centres	2,140	EMR	General	Yes	- Demographic questions (4)<br>- WTS questions (4)<br>- WTS by data type questions (15)<br>- Reasons why respondents are unwilling to share (1)	English	1x electronic survey distributed via email	NR	Future preferences	NR

*Research scenarios about: (1) evaluating drug costs for the elderly, (2) safety of breast implants, (3) whether clinicians were treating heart disease according to guidelines. To indicate a willingness to share, data sharing options included standard consent models (no consent, notification model, opt-out model, no use of the data permitted). †European heads of imaging departments (n=428) and speakers at the Clinical Trials in Radiology sessions at the European Congress of Radiology (n=32). ‡Paediatric brain tumour and controls, paediatric autism, adult and paediatric epilepsy, adult and paediatric liver cancer, adult pancreatic cancer. §Participants previously enrolled in a cancer genetics registry (that includes patients, a small percentage of first-

degree relatives, and controls) and self-referrals from the public. [¶]Oversampled African-Americans and Hispanics. [#]18 scenarios in total. Participants received 6 scenarios, each with different combinations of the three attributes and were asked to indicate their data-sharing preferences.

AI, Artificial Intelligence; ANZCTR, Australian New Zealand Clinical Trials Registry; Clin-epi, clinical-epidemiological; dbGaP, The database of Genotypes and Phenotypes; DCE, discrete choice experiment; DIRECT, Diabetes Research on Patient Stratification; EHR, electronic health record; EMR, electronic medical record; ENGAGE, European Network for Genetic and Genomic Epidemiology; FAIR, findability, accessibility, interoperability, and reusability; FGD, focus group discussion; GP, general practitioner; GWAS, genome-wide association studies; IADR, International Association for Dental Research; IPD, individual participant data; IT, information technology; KAP, knowledge, attitudes, practices; LD, Leukodystrophy; LMIC, Low- or Middle-Income Country; MH, mental health; ML, Machine learning; NA, not applicable; NHS, National Health Service; NR, not reported; NS, not specified; PCHR, personally controlled health records; PH, physical health; PIP, Participant Issues and Expectations Project; QDS, questionnaire development system; REC, research ethics committee; SHGP, Saudi Human Genome Program; WTS, willingness to share.
