# Supplementary material for: How do we measure data sharing in the biomedical sciences? A measurement systematic review of biomedical data sharing-related knowledge, attitudes and practices across stakeholder groups, data types and geographies
Source: BMJ Open. 2026 Mar 11;16(3):e100314. doi: 10.1136/bmjopen-2025-100314 (PMC12983744; doi:10.1136/bmjopen-2025-100314)
Supplement: Supplementary file 5 [file bmjopen-2025-100314.reviewer_comments.pdf]

PEER REVIEW HISTORY

BMJ Open publishes all reviews undertaken for accepted manuscripts. Reviewers are asked to complete a checklist review form and are provided with free text boxes to elaborate on their assessment. These free text comments are reproduced below.

ARTICLE DETAILS

Title (Provisional)

How do we measure data sharing in the biomedical sciences? A measurement systematic review of biomedical data sharing-related knowledge, attitudes and practices across stakeholder groups, data types, and geographies.

Authors

Maxwell, Lauren; Shreedhar, Priya; Gilyan, Regina; Naccache, Mirna; Terry, Robert F

VERSION 1 - REVIEW

**Reviewer**	**1**
**Name**	**Jean-Quartier, Claire**
**Affiliation**	**Graz University of Technology**
**Date**	**29-Mar-2025**
**COI**	**None**

The paper presents a systematic review on surveys of knowledge, attitude and practices (KAP) of biomedical data sharing based on quantitative survey instruments. It is comprehensibly written and well structured.

Please indicate in the manuscript why you excluded related research to keywords human and patient from your query? Furthermore, please mention why studies based on repositories were excluded? Such points could be indicated in as part of the methods but also discussed in the end.

It would be also interesting to have mean outcomes and results of the surveys presented and compared, also summarized visually or in a table other than table 1 (outline only for fast comparison) if applicable!

**Reviewer**	**2**
**Name**	**Naudet, Florian**
**Affiliation**	**University of Rennes**
**Date**	**06-Apr-2025**
**COI**	**None**

Florian Naudet, MD, PhD, Rennes University

This is an excellent and very interesting manuscript with many strengths. I applaud the preregistration and the very detailed reporting that follows the registered protocol as well as details about the few deviations.

I only have minor comments.

The abstract is well written but I would expect a few words about the main limitations.

INTRO/METHODS:

- This protocol paper should be referenced : https://open-research-europe.ec.europa.eu/articles/3-151.

I expect some comments about the links/ovelaps/differences between the submitted systematic review and the referenced scoping review by the same team.

- In "The search strategy was tailored to each database and piloted with assistance from an information

scientist." Please refer to the PRESS guideline (in JCE) if appropriate ;

- In "The heat maps in Figures 3 and 4 were created in R Studio version 2024.09.1+394." please cite all libraries that were used.

RESULTS :

- Flowchart : "Reports excluded" based on full text (please provide the number for the header : n=XXX) ;

- Figure 3 : please detail EMR in the legend ;

- Figure 4 : please detail AI and ERC in the legend ;

- Table 1 is likely big for the main manuscript and risks confusing the reader. However it is great : could it be moved to the appendix ;

- Table 3 should detail in its legend the meaning of the COSMIN ratings as the table should be possible to read independently of the text ;

- "All data are available in the publication and the related attachments." is not acceptable : a link to the OSF (or any other repository) should provide an access to the full dataset and the codes. The legend of this figures should also indicate that data and code to replicate the figure are available on XXXX with XXXX being the link to the chosen repository.

**Reviewer**	**3**
**Name**	**Markham, Sarah**
**Affiliation**	**King's College, London, Biostatistics**
**Date**	**16-Nov-2025**
**COI**	**None**

Methodologically and clinically relevant paper

VERSION 1 - AUTHOR RESPONSE

Reviewer: 1
Dr. Claire Jean-Quartier, Graz University of Technology

1. **The paper presents a systematic review on surveys of knowledge, attitude and practices (KAP) of biomedical data sharing based on quantitative survey instruments. It is comprehensibly written and well structured. Please indicate in the manuscript why you excluded related research to keywords human and patient from your query?**
   We applied best practice for excluding animal studies from human subject research. These strategies were developed by an external group. Please note the NOTs in the search strategy, as copied below. These NOTs ensure that animal studies are excluded while retaining human subjects research.

APA PsycInfo & CINAHL

#10	TI (((animal* OR canine* OR dog* or feline* OR hamster* OR lamb* OR mice OR mouse OR monkey* OR murine OR pig* OR piglet* OR porcine OR primate* OR rabbit* OR rat* OR rodent* OR sheep* OR frog* OR worm* OR trematode))) NOT TI (((human* OR patient*))))
#11	S9 NOT S10

2. **Furthermore, please mention why studies based on repositories were excluded? Such points could be indicated in as part of the methods but also discussed in the end.**

We thank the reviewer for catching this. This represents an error in the text. Rather than "repositories" we meant "repositories of samples" and have now changed this to "sample collections," which is a more widely accepted term for sample collections that do not qualify as biobanks.

3. **It would be also interesting to have mean outcomes and results of the surveys presented and compared, also summarized visually or in a table other than table 1 (outline only for fast comparison) if applicable!**

Because of the heterogeneity in research questions, populations, survey domains, etc., and because a comparison of the outcomes was not in scope for this measurement systematic review, we decided not to report on the outcomes from included studies. Please see this statement from our

Strengths and Limitations section: *Lastly, we did not compare the findings of the quantitative surveys because of the high level of heterogeneity in survey objectives and design and because that was out of scope for this measurement systematic review.*

For these reasons, we are unable to include findings from individual studies in the text and tables of this manuscript.

Reviewer: 2
Dr. Florian Naudet, University of Rennes

1. **This is an excellent and very interesting manuscript with many strengths. I applaud the preregistration and the very detailed reporting that follows the registered protocol as well as details about the few deviations. I only have minor comments. The abstract is well written but I would expect a few words about the main limitations.**

We thank the reviewer for their positive feedback. We initially added text to the abstract to address the main limitations. The additional text was as follows: "This is the first systematic comparison of quantitative instruments assessing data-reuse KAP and structured evaluation using COSMIN. Limitations include limited access to 11 survey instruments, sparse reporting of survey development or validation activities, inability to assess most COSMIN domains, and searches for updates conducted only in Ovid (Medline) due to funding constraints."

We subsequently removed this text because we were not able to meet the requirement for restructuring the abstract (see request, above) and keeping the abstract to 300 words. We hope the reviewer understands this decision. We tried several approaches, but could not include the information on limitations without exceeding the word limit.

2. **INTRO/METHODS:**
- This protocol paper should be referenced : https://open-research-europe.ec.europa.eu/articles/3-151. I expect some comments about the links/ovelaps/differences between the submitted systematic review and the referenced scoping review by the same team.

Firstly, and most importantly, we would like to thank the reviewer for taking the time to review our work on this manuscript and our work on the protocol for a different review, *"How do we measure the costs, benefits, and harms of sharing data from biomedicalstudies? A protocol for a scoping review."* The reviewer's insightful comments have strengthened both manuscripts. As it is increasingly difficult to find reviewers willing to volunteer their time for peer review, leading to important delays in the communication of scientific evidence, the reviewer' s contributions are especially appreciated by our team.

These two reviews have different research questions and approaches. The current review takes a measurement systematic review approach wherein we apply the COSMIN criteria for an in-depth exploration of the methodological rigour of quantitative survey development, the focus of this systematic review, *“How do we measure data sharing in the biomedicalsciences? A measurement systematic review of biomedicaldata sharing-related knowledge, attitudes and practices across stakeholder groups, data types, and geographies.”*

The protocol cited by the reviewer refers to a separate review intended to summarise existing evidence on how we measure the costs, benefits, and harms associated with the reuse of data and samples from biomedical research. We chose a scoping review rather than a systematic review to provide a broad summary of related research, including qualitative approaches, health economic studies, and bibliometric research.

These two projects employ different methodologies, target distinct conceptual domains, and address non-overlapping research questions. The scoping review protocol focuses on identifying how the literature characterises and measures *costs, benefits, and harms of data sharing*. In contrast, the measurement systematic review evaluates the *development, validity, and measurement properties* of quantitative instruments assessing *knowledge, attitudes, and practices* related to data sharing. Because the two reviews do not draw upon one another analytically, conceptually, or methodologically, and because the scoping reviewdoes not inform the search strategy, eligibility criteria, data extraction, or interpretation of findings in the measurement systematic review, referencing the protocol would not provide additional context or justification for the measurement review.

While our research team is leading both studies, which broadly relate to data reuse in biomedical research, the approaches and focuses are very different, so we did not reference the scoping review protocol in the results of the measurement systematic review. We hope that this outcome is acceptable to the reviewer and again thank them for taking the time to review and provide valuable feedback on both manuscripts from our research team. We feel this approach best preserves methodological clarity and avoids implying a conceptual linkage that was not part of the study design. We greatly appreciate your close reading of both manuscripts and your consistently constructive suggestions, and we hope this clarification addresses your concern.

3. **In "The search strategy was tailored to each database and piloted with assistance from an information scientist." Please refer to the PRESS guideline (in JCE) if appropriate;**

We have included the following text to address the reviewer's comment here: "In keeping with the Peer Review of Electronic Search Strategies (PRESS) 2015 Guidelines [20], the systematic search strategy was developed through translating the research questions into a Patient or Problem, Intervention, Comparison, and Outcome (PICO) format, using Boolean and proximity operators, combining related MeSH terms, where available, and free-text terms, applying limits and filters, including a filter for animal studies, tailoring the search to each database, and pil oting the search to ensure appropriate syntax. The search strategies were reviewed by an information scientist at Universitätsklinikum Heidelberg Library."

4. **In "The heat maps in Figures 3 and 4 were created in R Studio version 2024.09.1+394." please cite all libraries that were used.**

We have cited the specific R Libraries. The corresponding text now reads: "The heat maps in Figures 3 and 4 were created with the 'tidyverse' library in R Studio version 2024.09.1+394."

5. **RESULTS :**
   - **Flowchart : "Reports excluded" based on full text (please provide the number for the header : n=XXX) ;**
     We have updated the flowchart to include the total number of reports excluded.
   - **Figure 3 : please detail EMR in the legend**
     We have included the definition of EMR in the legend.
   - **Figure 4 : please detail AI and ERC in the legend**
     We have included the definitions of AI and ERC in the legend.

6. **Table 1 is likely big for the main manuscript and risks confusing the reader. However it is great : could it be moved to the appendix ;**

We have moved Table 1 to the Appendix.

7. **Table 3 should detail in its legend the meaning of the COSMIN ratings as the table should be possible to read independently of the text ;**

We have amended the Table 3 legend (now Table 2) to describe the COSMIN ratings.

8. **"All data are available in the publication and the related attachments." is not acceptable : a link to the OSF (or any other repository) should provide an access to the full dataset and the codes. The legend of this figures should also indicate that data and code to replicate the figure are available on XXXX with XXXX being the link to the chosen repository.**

No data were extracted from publications other than the data in the manuscript tables. We have
uploaded the R code for the heat maps to GitHub and referenced this in the manuscript.

Reviewer: 3
Dr. Sarah Markham, King's College, London, Birkbeck University of London Comments to the Author:

Methodologically and clinically relevant paper

We thank the reviewer for their positive feedback.
